# All-Cause and Cause-Specific Mortality in Patients With Bipolar II Disorder

**DOI:** 10.1001/jamanetworkopen.2026.5535

**Published:** 2026-04-07

**Authors:** Chih-Wei Hsu, Yang-Chieh Brian Chen, Edward Chia-Cheng Lai, Andrew A. Nierenberg, Michael Berk, Sheng-Yu Lee, Liang-Jen Wang, Mu-Hong Chen, Yao-Hsu Yang, Chih-Sung Liang, Andre F. Carvalho

**Affiliations:** 1 Department of Psychiatry, Kaohsiung Chang Gung Memorial Hospital and Chang Gung University College of Medicine, Kaohsiung, Taiwan; 2 Department of Psychiatry and Behavioral Sciences, The University of Texas Health Science Center at Houston, Houston; 3 School of Pharmacy, Institute of Clinical Pharmacy and Pharmaceutical Sciences, College of Medicine, National Cheng Kung University, Tainan, Taiwan; 4 Population Health Data Center, National Cheng Kung University, Tainan, Taiwan; 5 Dauten Family Center for Bipolar Treatment Innovation, Massachusetts General Hospital, Harvard Medical School, Boston; 6 IMPACT, The Institute for Mental and Physical Health and Clinical Translation, School of Medicine, Barwon Health, Deakin University, Geelong, Victoria, Australia; 7 Department of Psychiatry, Kaohsiung Veterans General Hospital, Kaohsiung, Taiwan; 8 Department of Psychiatry, Faculty of Medicine, Kaohsiung Medical University, Kaohsiung, Taiwan; 9 Department of Child and Adolescent Psychiatry, Kaohsiung Chang Gung Memorial Hospital, Chang Gung University College of Medicine, Kaohsiung, Taiwan; 10 Department of Psychiatry, Taipei Veterans General Hospital, Taipei, Taiwan; 11 Department of Psychiatry, College of Medicine, National Yang Ming Chiao Tung University, Taipei, Taiwan; 12 Department of Traditional Chinese Medicine, Chiayi Chang Gung Memorial Hospital, Chiayi, Taiwan; 13 Health Information and Epidemiology Laboratory of Chang Gung Memorial Hospital, Chiayi, Taiwan; 14 School of Traditional Chinese Medicine, College of Medicine, Chang Gung University, Taoyuan, Taiwan; 15 Department of Psychiatry, Beitou Branch, Tri-Service General Hospital, National Defense Medical University, Taipei, Taiwan; 16 Department of Psychiatry, National Defense Medical University, Taipei, Taiwan

## Abstract

**Importance:**

Whether bipolar II disorder (BD-II) is associated with increased long-term mortality remains uncertain because most studies have not distinguished BD-II from bipolar I disorder (BD-I).

**Objective:**

To examine whether BD-II is associated with elevated all-cause and cause-specific mortality compared with population controls, unaffected siblings, and those with BD-I.

**Design, Setting, and Participants:**

This population-based, retrospective cohort study used data from Taiwan’s National Health Insurance Database from January 1, 2000, to December 31, 2022. Individuals 12 years or older with 2 or more psychiatrist-assigned BD-II diagnoses were individually matched to 4 controls without BD-II by sex and birthdate. Additional comparisons included unaffected biological siblings and a BD-I cohort. Follow-up was from the index date to death or December 31, 2022. Data were analyzed from June to August 2025.

**Exposures:**

Clinical diagnosis of BD-II.

**Main Outcomes and Measures:**

Primary outcome was all-cause mortality; secondary outcomes were natural-cause and unnatural-cause mortality. Cox proportional hazards regression models estimated adjusted hazard ratios (AHRs) with 95% CIs, controlling for age, sex, income, urbanization, health care use, and comorbidity.

**Results:**

The study included 11 427 individuals with BD-II (mean [SD] age, 39.6 [16.6] years; 7073 [61.9%] female) and 45 708 matched controls (mean [SD], 39.6 [16.7] years; 28 292 [61.9%] female). During a mean (SD) follow-up of 7.3 (5.1) years, 1089 patients with BD-II and 1879 controls died (AHR, 1.62; 95% CI, 1.47-1.78). Excess mortality in BD-II was observed for natural causes (AHR, 1.37; 95% CI, 1.23-1.52) and for unnatural causes (AHR, 4.46; 95% CI, 3.53-5.64). Natural-cause deaths included mental and behavioral disorders; circulatory, respiratory, digestive, and skin or subcutaneous diseases; and symptoms, signs, and abnormal clinical and laboratory findings not elsewhere classified. Unnatural-cause deaths were predominantly unintentional injuries, suicide, and assault or homicide. Findings were consistent across sex, age, and psychiatric comorbidities. In within-family analyses, BD-II remained associated with higher all-cause (AHR, 1.31; 95% CI, 1.00-1.72) and unnatural-cause mortality (AHR, 2.05; 95% CI, 1.43-2.95) but not natural-cause mortality. Compared with BD-I, BD-II had higher all-cause (AHR, 1.24; 95% CI, 1.01-1.53) and natural-cause mortality (AHR, 1.45; 95% CI, 1.14-1.86) but not unnatural-cause mortality.

**Conclusions and Relevance:**

In this cohort study, BD-II was associated with a significant risk of premature mortality across multiple causes; even after accounting for shared familial factors and relative to BD-I, all-cause mortality remained elevated. These findings underscore the importance of comprehensive psychiatric care for individuals with BD-II.

## Key Points

**Question:**

Is bipolar II disorder associated with increased long-term mortality risk?

**Findings:**

In this cohort study of 11 427 participants with bipolar II disorder, patients had higher all-cause mortality risk compared with individuals without bipolar II disorder. Participants with bipolar II disorder had an increased mortality from both natural causes and unnatural causes, such as suicide.

**Meaning:**

This study’s results suggest the need for integrated psychiatric care and targeted suicide prevention for people with bipolar II disorder.

## Teaser

This cohort study of individuals with bipolar II disorder examines the association of bipolar II disorder with elevated all-cause or cause-specific mortality.

## Introduction

Bipolar II disorder (BD-II) is a bipolar subtype first proposed in the 1970s and codified in the *Diagnostic and Statistical Manual of Mental Disorders, Fourth Edition*.^1,2^ A diagnosis of BD-II requires at least 1 major depressive episode and 1 hypomanic episode, with no lifetime history of mania.^1^ Accordingly, BD-II involves elevated mood states that are less intense and generally briefer than the full manic episodes characteristic of bipolar I disorder (BD-I); hypomania, by definition, lacks the marked functional impairment or psychotic features that can accompany mania.^1^ Clinically, BD-II is distinguished by more frequent and longer depressive episodes than BD-I,^3,4^ whereas hypomanic phases are relatively mild. Contrary to the generalization of hypomania to mania in BD-II as milder, patients experience substantial depressive burden and functional impairment,^5,6^ indicating that BD-II should not be regarded as simply a milder form of BD. Epidemiologically, lifetime prevalence is approximately 0.4% in the general population,^7^ and diagnoses are more common in females.^8^

BD overall is associated with excess mortality from both natural and unnatural causes. Meta-analytic evidence suggests that all-cause mortality is approximately doubled, with particularly high relative risks for suicide (12-fold) and other unnatural causes (7-fold) alongside a substantial increase in natural-cause deaths (2-fold).^9^ However, most mortality studies have not separated BD-II from BD-I, leaving BD-II’s specific mortality profile uncertain. Limited data indicate substantial premature mortality in BD-II, for example, an estimated 10- to 20-year reduction in life expectancy,^1^ with cardiovascular disease a major contributor to natural-cause deaths.^10,11^ This is likely predicated by shared risk factors and operative biological pathways as well as adverse effects of prescribed agents.^12^ Unnatural causes, especially suicide, also appear elevated. A large Swedish registry^13^ reported more frequent suicide attempts in BD-II than BD-I, yet meta-analytic work^14^ suggests that the risk of suicide death is broadly similar across the 2 subtypes. Many BD-II–focused studies have been underpowered or have not analyzed BD-II separately,^11,15,16,17^ rendering conclusions tentative. Whether BD-II differs from BD-I in all-cause or cause-specific mortality remains largely unresolved because direct comparisons are scarce.^18^

Given this paucity of definitive data, we hypothesized that BD-II is associated with elevated all-cause or cause-specific mortality. The current study addresses this gap by quantifying mortality in BD-II relative to matched population controls, unaffected siblings, and BD-I to delineate BD-II–specific risk profiles.

## Methods

This cohort study adhered to the Strengthening the Reporting of Observational Studies in Epidemiology (STROBE) reporting guideline. Approval was obtained from the institutional review board of Chang Gung Memorial Hospital, which waived the need for informed consent because the patient data used in the study were deidentified and encrypted. Detailed procedures are given in the eMethods in Supplement 1.

### Study Cohorts

We used Taiwan’s National Health Insurance Research Database (NHIRD), a nationwide claims repository (>99% population coverage) containing deidentified, linkable beneficiary records for demographics and complete outpatient and inpatient encounters with *International Classification of Diseases, Ninth Revision, Clinical Modification (ICD-9-CM)*– and *International Statistical Classification of Diseases, Tenth Revision, Clinical Modification (ICD-10-CM)*–coded diagnoses (*ICD-9-CM* through 2015; *ICD-10-CM* thereafter), procedures, and dispensed prescriptions. A unique encrypted identifier enables deterministic linkage to the National Death Registry for exact dates and certified causes of death.^17,19^ We analyzed claims made from January 1, 2000, to December 31, 2022.

The BD-II cohort included individuals who received 2 or more psychiatrist-assigned diagnoses of BD-II (*ICD-9-CM* code 296.89; *ICD-10-CM* code F31.81) between January 1, 2001, and December 31, 2021. We excluded persons with unspecified sex or birth date and anyone with any BD-I diagnosis recorded throughout the entire database period (*ICD-9-CM* codes 296.0, 296.1, 296.4–296.7; *ICD-10-CM* codes F30.1-F30.4, F30.9, F31.0-F31.7, F31.89). To enhance diagnostic specificity, we restricted the study to participants 12 years or older at the index BD-II diagnosis.^20^ To contextualize mortality risk, we formed a population-matched control cohort: for each patient with BD-II, 4 NHIRD beneficiaries with no BD-II across their claims history were randomly selected and individually matched on sex and birth date (±6 months). Two additional comparators were assembled. First, we constructed an unaffected sibling cohort from patients with BD-II who had 1 or more siblings identifiable in the database (beneficiaries sharing ≥1 biological parent). We included the index case and all biologically related siblings with no diagnosis of any bipolar disorder, enabling within-family contrasts that minimize confounding by shared familial and early-life environmental factors. Second, a BD-I cohort was constructed using criteria analogous to those for BD-II, identifying patients with BD-I during 2001 to 2021 for cross-subtype comparisons. The study workflow and eligibility are shown in eFigure 1 in Supplement 1.

Cohort-specific index dates were as follows: (1) matched controls (the first BD-II diagnosis date for the patients with BD-II [applied to the matched set]); (2) unaffected siblings (January 1, 2001, for both patients with BD-II and siblings [earliest date with comprehensive coverage]); and (3) BD-I (first BD-II diagnosis for patients with BD-II and first BD-I diagnosis for patients with BD-I). All participants were followed up from their assigned index date until death or December 31, 2022, whichever occurred first.

### Outcomes and Covariates

The primary outcome was all-cause mortality. Secondary outcomes were cause-specific mortality grouped by *ICD-9-CM* or *ICD-10-CM* chapter into natural causes (all nonexternal causes) and unnatural causes (external causes), with unnatural deaths subtyped as unintentional injury, suicide, or assault or homicide; deaths with missing or nonclassifiable codes were categorized as unknown. Baseline covariates included sex; age at cohort entry; income quartile (database-wide proxy for socioeconomic status: >75th, 50th-75th, 25th-50th, or ≤25th); residential urbanization (4-tier scale from most to least urbanized)^21^; psychiatric health care use (number of psychiatric hospitalizations and outpatient visits); medical comorbidity burden using the Charlson Comorbidity Index (CCI)^22^; 7 psychiatric comorbidity groups selected for known mortality associations: neurodevelopmental disorders,^23^ anxiety disorders,^24^ obsessive-compulsive disorder,^25^ posttraumatic stress disorder,^26^ eating disorders,^27^ substance use disorders,^28^ and personality disorders^29^; and psychiatric medication use (antidepressants, antipsychotics, and mood stabilizers). Comprehensive *ICD-9-CM* or *ICD-10-CM* code and psychiatric medication lists appear in eTables 1 to 3 in Supplement 1.

### Statistical Analysis

Baseline characteristics were summarized as numbers (percentages) for categorical variables and means (SDs) for continuous variables. Survival was visualized with Kaplan-Meier curves; differences between groups were assessed using the log-rank test. Cox proportional hazards regression models estimated hazard ratios (HRs) with 95% CIs, using time since cohort entry as the time scale. Analyses were first conducted for all-cause mortality and then repeated for each cause-specific outcome. Model 1 reported crude HRs; model 2 additionally adjusted for sex, birth year, income quartile, urbanization level, psychiatric health care use, and CCI and reported adjusted HRs (AHRs). Psychiatric medication use was reported descriptively but not included as a covariate. Missing covariate values were coded as unknown categories and retained in all models.

Prespecified secondary analyses included the following: (1) 2 sensitivity analyses repeating models 1 to 2, with the first in a complete-case sample excluding participants with missing covariates and the second reincluding patients with diagnostic conversion from BD-II to BD-I^30^; (2) subgroup analyses stratified by sex, age (adolescents, adults, or older adults), and each psychiatric comorbidity group; and (3) comparator analyses contrasting patients with BD-II who had 1 or more siblings vs their unaffected siblings and BD-II vs BD-I. The same modeling framework (models 1-2) was applied to comparator analyses; for the sibling comparison, model 2 additionally adjusted for sibling birth order and used cluster-robust (sandwich) SEs to account for within-family correlation.^31^ All analyses were performed in SAS, version 9.4 (SAS Institute Inc). Statistical significance was set at 2-sided *P* < .05, corresponding to 95% CIs excluding 1.00. Analysis was performed from June to August 2025.

## Results

### Matched Population Analyses

Table 1 summarizes baseline characteristics for the 11 427 patients with BD-II (mean [SD] age, 39.6 [16.6] years; 7073 [61.9%] female and 4354 [38.1%] male) and their 45 708 matched controls (mean [SD] age, 39.6 [16.7] years; 28 292 [61.9%] female and 17 416 [38.1%] male) identified between 2001 and 2021. The mean (SD) follow-up was 7.3 (5.1) years. Compared with controls, patients with BD-II were more likely to be in the lower half of the income distribution (≤50th percentile: 6753 of 11 427 [59.1%] vs 20 855 of 45 708 [45.7%]) and have a greater medical comorbidity burden (CCI ≥1: 3977 of 11 427 [34.8%] vs 8693 of 45 708 [19.0%]) and psychiatric comorbidities (eg, anxiety disorders: 2536 of 11 427 [22.2%] vs 569 of 45 708 [1.2%]). During follow-up, 1089 patients and 1879 controls died, corresponding to crude mortality rates of 13.6 and 5.6 deaths per 1000 person-years, respectively. Kaplan-Meier curves diverged early and remained separated throughout follow-up for all-cause, natural-cause, and unnatural-cause mortality (eFigure 2 in Supplement 1).

**Table 1.** Characteristics of All Participants With Bipolar II Disorder

Characteristic	No. (%) of participants^a^	
	Case group (n = 11 427)	Control group (n = 45 708)
Age, mean (SD), y	39.6 (16.6)	39.6 (16.7)
Sex		
Female	7073 (61.9)	28 292 (61.9)
Male	4354 (38.1)	17 416 (38.1)
Personal income level		
>25th (Highest)	1845 (16.1)	10 953 (24.0)
25th-50th	2208 (19.3)	10 546 (23.1)
50th-75th	2604 (22.8)	10 358 (22.7)
≤75th (Lowest)	4149 (36.3)	10 497 (23.0)
Unknown	621 (5.4)	3354 (7.3)
Personal urbanization level		
1 (Urban)	6520 (57.1)	25 039 (54.8)
2	4010 (35.1)	16 410 (35.9)
3	680 (6.0)	3057 (6.7)
4 (Rural)	118 (1.0)	395 (0.9)
Unknown	99 (0.9)	807 (1.8)
No. of psychiatric hospitalizations		
0	10 317 (90.3)	45 633 (99.8)
1	727 (6.4)	51 (0.1)
≥2	383 (3.4)	24 (0.1)
No. of psychiatric outpatient visits		
0-6	7125 (62.4)	44 961 (98.4)
7-13	2421 (21.2)	526 (1.2)
≥14	1881 (16.5)	221 (0.5)
No. of medical comorbidities (Charlson Comorbidity Index)		
0	7450 (65.2)	37 015 (81.0)
1-2	3030 (26.5)	7099 (15.5)
≥3	947 (8.3)	1594 (3.5)
Psychiatric comorbidities		
Neurodevelopmental disorders	348 (3.0)	76 (0.2)
Anxiety disorders	2536 (22.2)	569 (1.2)
Obsessive-compulsive disorder	198 (1.7)	38 (0.1)
Posttraumatic stress disorder	89 (0.8)	8 (<0.1)
Eating disorders	109 (1.0)	13 (<0.1)
Substance use disorders	561 (4.9)	38 (0.1)
Personality disorders	217 (1.9)	9 (<0.1)
Psychiatric medication use		
Antidepressant use	813 (7.1)	30 (0.1)
Antipsychotic use	860 (7.5)	69 (0.2)
Mood stabilizer use	624 (5.5)	26 (0.1)

^a^ Unless otherwise indicated.

**Figure 1.**
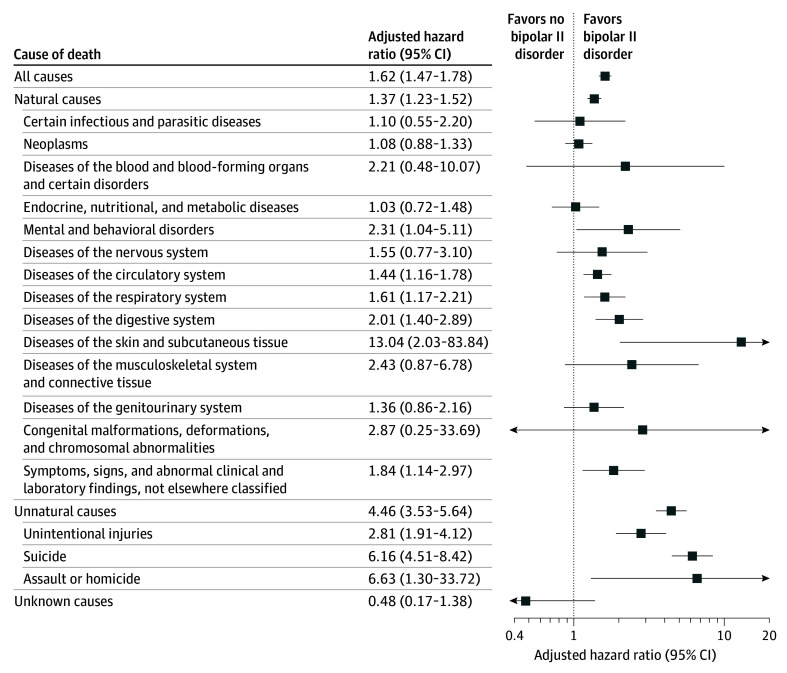
Forest Plot of All-Cause and Cause-Specific Mortality in Bipolar II Disorder vs Matched Population Controls Hazard ratios were adjusted for sex, birth year, income quartile, urbanization level, psychiatric health care use, and Charlson Comorbidity Index.

Both crude (model 1) and adjusted (model 2) estimates showed elevated mortality in BD-II. For all-cause mortality, the HR for model 1 was 2.44 (95% CI, 2.27-2.63), and the AHR for model 2 was 1.62 (95% CI, 1.47--1.78). Mortality from natural causes was increased (model 1: HR, 1.99; 95% CI, 1.83-2.16; model 2: AHR, 1.37; 95% CI, 1.23-1.52), as was mortality from unnatural causes (model 1: HR, 7.23; 95% CI, 5.98-8.76; model 2: AHR, 4.46; 95% CI, 3.53-5.64) (Table 2 and Figure 1). By contrast, mortality attributed to unknown causes did not differ significantly (model 1: HR, 1.22; 95% CI, 0.56-2.68; model 2: AHR, 0.48; 95% CI, 0.17-1.38). Within natural-cause categories, risks were higher across several *ICD-9-CM* and *ICD-10-CM* chapters, including mental and behavioral disorders; circulatory system diseases; respiratory system diseases; digestive system diseases; diseases of the skin and subcutaneous tissue; and symptoms, signs and abnormal clinical and laboratory findings, not elsewhere classified. For unnatural causes, risks were also increased for unintentional injuries (model 2: AHR, 2.81; 95% CI, 1.91-4.12), suicide (model 2: AHR, 6.16; 95% CI, 4.51-8.42), and assault or homicide (model 2: AHR, 6.63; 95% CI, 1.30-33.72).

**Table 2.** Risk of All-Cause and Cause-Specific Mortality in Participants With Bipolar II Disorder and Matched Controls

Characteristic	No. (%) of participants		HR (95% CI) for model 1
	Case group (n = 11 427)	Control group (n = 45 708)	
All causes	1089 (9.5)	1879 (4.1)	2.44 (2.27-2.63)
Natural causes	793 (6.9)	1685 (3.7)	1.99 (1.83-2.16)
Certain infectious and parasitic diseases	19 (0.2)	39 (0.1)	2.04 (1.18-3.54)
Neoplasms	174 (1.5)	551 (1.2)	1.33 (1.12-1.58)
Diseases of the blood and blood-forming organs and certain disorders	4 (<0.1)	5 (<0.1)	3.31 (0.89-12.34)
Endocrine, nutritional, and metabolic diseases	68 (0.6)	145 (0.3)	1.98 (1.48-2.64)
Mental and behavioral disorders	21 (0.2)	23 (0.1)	3.88 (2.15-7.01)
Diseases of the nervous system	22 (0.2)	38 (0.1)	2.46 (1.45-4.16)
Diseases of the eye and adnexa	0	0	NA
Diseases of the ear and mastoid process	0	0	NA
Diseases of the circulatory system	197 (1.7)	429 (0.9)	1.94 (1.64-2.30)
Diseases of the respiratory system	108 (0.9)	176 (0.4)	2.60 (2.04-3.30)
Diseases of the digestive system	77 (0.7)	106 (0.2)	3.05 (2.28-4.09)
Diseases of the skin and subcutaneous tissue	4 (<0.1)	2 (<0.1)	8.34 (1.53-45.51)
Diseases of the musculoskeletal system and connective tissue	10 (0.1)	11 (<0.1)	3.82 (1.62-9.00)
Diseases of the genitourinary system	36 (0.3)	84 (0.2)	1.81 (1.23-2.68)
Pregnancy, childbirth, and the puerperium	0	2 (<0.1)	NA
Certain conditions originating in the perinatal period	0	0	NA
Congenital malformations, deformations, and chromosomal abnormalities	1 (<0.1)	2 (<0.1)	2.11 (0.19-23.30)
Symptoms, signs, and abnormal clinical and laboratory findings, not elsewhere classified	52 (0.5)	72 (0.2)	3.04 (2.13-4.35)
Unnatural causes	288 (2.5)	166 (0.4)	7.23 (5.98-8.76)
Unintentional injuries	81 (0.7)	85 (0.2)	3.99 (2.95-5.41)
Suicide	203 (1.8)	78 (0.2)	10.82 (8.33-14.05)
Assault or homicide	4 (<0.1)	3 (<0.1)	5.47 (1.23-24.46)
Unknown causes	8 (0.1)	28 (0.1)	1.22 (0.56-2.68)

Abbreviations: AHR, adjusted hazard ratio; HR, hazard ratio; NA, not applicable.

Both sensitivity analyses yielded results consistent with the primary findings (eTables 4 and 5 in Supplement 1): the first excluded participants with missing covariate data, and the second reincluded 2164 patients (15.9% of all patients initially identified with BD-II) who had diagnostic conversion from BD-II to BD-I. In sex-stratified analyses (model 2), females and males with BD-II had comparably elevated risks of all-cause mortality (female: AHR, 1.58; 95% CI, 1.37-1.83; male: AHR, 1.66; 95% CI, 1.46-1.88) and natural-cause mortality (female: AHR, 1.31; 95% CI, 1.11-1.54; male: AHR, 1.43; 95% CI, 1.24-1.64) relative to their unaffected counterparts. In contrast, the relative risk of unnatural-cause death was higher in females (AHR, 5.41; 95% CI, 3.74-7.83) than in males (AHR, 4.01; 95% CI, 2.95-5.44) (eTables 6 and 7 in Supplement 1). Age-stratified models (model 2) showed a gradient: relative to matched controls, all-cause mortality AHRs were numerically highest in adolescents (AHR, 3.49; 95% CI, 1.01-12.03), intermediate in adults (AHR, 1.95; 95% CI, 1.72-2.21), and lower yet still higher in older adults (AHR, 1.31; 95% CI, 1.13-1.51). Similar patterns were seen for cause-specific outcomes, with higher estimates in adults than older adults for both natural causes (adults: AHR, 1.45; 95% CI, 1.25-1.68; older adults: AHR, 1.33; 95% CI, 1.14-1.55) and unnatural causes (adults: AHR, 5.21; 95% CI, 4.03-6.74; older adults: AHR, 1.38; 95% CI, 0.63-2.99) (eTables 8-10 in the Supplement). Sequential adjustment for psychiatric comorbidity groups (model 2) produced estimates broadly similar to the main analyses (Table 3). For all-cause mortality, AHRs ranged from 1.58 after adjusting for substance use disorders to 1.63 after adjusting for anxiety disorders; for natural-cause mortality, from 1.35 (substance use disorders) to 1.40 (anxiety disorders); and for unnatural causes, from 4.30 (substance use disorders) to 4.53 (neurodevelopmental disorders).

**Table 3.** Risk of All-Cause and Cause-Specific Mortality in Participants With Bipolar II Disorder and Matched Controls by Psychiatric Comorbidity Group^a^

Characteristic	Adjusted hazard ratio (95% CI)^b^						
	Neurodevelopmental disorders	Anxiety disorder	Obsessive-compulsive disorder	Posttraumatic stress disorder	Eating disorder	Substance use disorder	Personality disorder
All causes	1.61 (1.47-1.77)	1.63 (1.48-1.79)	1.62 (1.47-1.78)	1.61 (1.47-1.77)	1.62 (1.47-1.78)	1.58 (1.44-1.74)	1.61 (1.47-1.77)
Natural causes	1.37 (1.23-1.52)	1.40 (1.25-1.55)	1.37 (1.24-1.53)	1.37 (1.23-1.52)	1.37 (1.23-1.52)	1.35 (1.21-1.50)	1.37 (1.23-1.52)
Certain infectious and parasitic diseases	1.10 (0.55-2.21)	1.10 (0.55-2.22)	1.10 (0.55-2.21)	1.08 (0.54-2.16)	1.10 (0.55-2.20)	1.06 (0.52-2.13)	1.10 (0.55-2.20)
Neoplasms	1.08 (0.88-1.33)	1.10 (0.90-1.36)	1.09 (0.88-1.33)	1.08 (0.88-1.33)	1.08 (0.88-1.33)	1.06 (0.86-1.30)	1.08 (0.88-1.33)
Diseases of the blood and blood-forming organs and certain disorders	2.22 (0.49-10.12)	2.04 (0.43-9.74)	2.22 (0.49-10.11)	2.21 (0.48-10.09)	2.21 (0.48-10.08)	2.24 (0.49-10.20)	2.22 (0.49-10.12)
Endocrine, nutritional, and metabolic diseases	1.03 (0.72-1.48)	1.07 (0.75-1.55)	1.03 (0.72-1.49)	1.03 (0.72-1.48)	1.03 (0.72-1.48)	1.03 (0.72-1.48)	1.03 (0.71-1.47)
Mental and behavioral disorders	2.26 (1.02-5.01)	2.30 (1.03-5.14)	2.30 (1.04-5.09)	2.31 (1.04-5.11)	2.31 (1.04-5.11)	2.31 (1.04-5.13)	2.28 (1.03-5.07)
Diseases of the nervous system	1.53 (0.77-3.07)	1.55 (0.77-3.12)	1.55 (0.77-3.11)	1.55 (0.77-3.10)	1.55 (0.77-3.10)	1.53 (0.76-3.07)	1.55 (0.77-3.10)
Diseases of the circulatory system	1.44 (1.16-1.78)	1.43 (1.15-1.77)	1.44 (1.16-1.78)	1.44 (1.16-1.78)	1.44 (1.16-1.78)	1.42 (1.15-1.76)	1.44 (1.16-1.78)
Diseases of the respiratory system	1.60 (1.17-2.20)	1.65 (1.20-2.27)	1.61 (1.17-2.21)	1.61 (1.17-2.21)	1.61 (1.17-2.21)	1.61 (1.17-2.20)	1.61 (1.17-2.21)
Diseases of the digestive system	2.01 (1.39-2.89)	2.06 (1.43-2.98)	2.02 (1.40-2.91)	2.01 (1.40-2.90)	2.01 (1.40-2.90)	1.85 (1.28-2.68)	2.00 (1.39-2.88)
Diseases of the skin and subcutaneous tissue	13.05 (2.03-83.86)	14.69 (2.26-95.30)	13.05 (2.03-83.90)	13.05 (2.03-83.86)	13.04 (2.03-83.84)	13.05 (2.03-83.90)	13.05 (2.03-83.87)
Diseases of the musculoskeletal system and connective tissue	2.44 (0.87-6.79)	2.59 (0.92-7.27)	2.45 (0.88-6.83)	2.43 (0.87-6.78)	2.43 (0.87-6.77)	2.38 (0.85-6.64)	2.46 (0.89-6.85)
Diseases of the genitourinary system	1.34 (0.85-2.12)	1.48 (0.94-2.34)	1.37 (0.87-2.17)	1.37 (0.86-2.16)	1.37 (0.86-2.16)	1.36 (0.86-2.15)	1.37 (0.86-2.16)
Congenital malformations, deformation, and chromosomal abnormalities	2.94 (0.25-34.40)	3.15 (0.27-36.73)	2.92 (0.25-34.11)	2.89 (0.25-33.79)	2.87 (0.25-33.69)	2.94 (0.25-34.47)	2.89 (0.25-33.92)
Symptoms, signs, and abnormal clinical and laboratory findings, not elsewhere classified	1.83 (1.14-2.95)	1.83 (1.13-2.96)	1.84 (1.14-2.97)	1.84 (1.14-2.97)	1.84 (1.14-2.97)	1.80 (1.12-2.91)	1.83 (1.13-2.95)
Unnatural causes	4.53 (3.58-5.73)	4.39 (3.46-5.57)	4.47 (3.53-5.65)	4.46 (3.52-5.64)	4.46 (3.52-5.64)	4.30 (3.40-5.45)	4.43 (3.50-5.61)
Unintentional injuries	2.83 (1.93-4.16)	2.67 (1.81-3.95)	2.81 (1.92-4.13)	2.81 (1.91-4.12)	2.80 (1.91-4.11)	2.61 (1.77-3.85)	2.79 (1.90-4.09)
Suicide	6.27 (4.59-8.58)	6.15 (4.49-8.43)	6.17 (4.51-8.44)	6.16 (4.50-8.42)	6.16 (4.50-8.42)	6.01 (4.39-8.23)	6.13 (4.48-8.38)
Assault or homicide	6.74 (1.33-34.25)	6.30 (1.20-33.23)	6.67 (1.31-33.93)	6.64 (1.30-33.81)	6.64 (1.30-33.81)	5.59 (1.03-30.24)	6.67 (1.31-33.93)
Unknown causes	0.48 (0.17-1.39)	0.42 (0.15-1.22)	0.48 (0.17-1.40)	0.48 (0.17-1.39)	0.48 (0.17-1.39)	0.47 (0.16-1.36)	0.48 (0.17-1.39)

^a^ Diseases of the eye and adnexa; diseases of the ear and mastoid process; pregnancy, childbirth, and the puerperium; or certain conditions originating in the perinatal period were not reported because no events occurred.

^b^ Hazard ratios were adjusted for all variables (sex, birth year, income level, urbanization level, psychiatric health care use, and Charlson Comorbidity Index).

### Unaffected Sibling and Cross-Subtype Analyses

**Figure 2.**
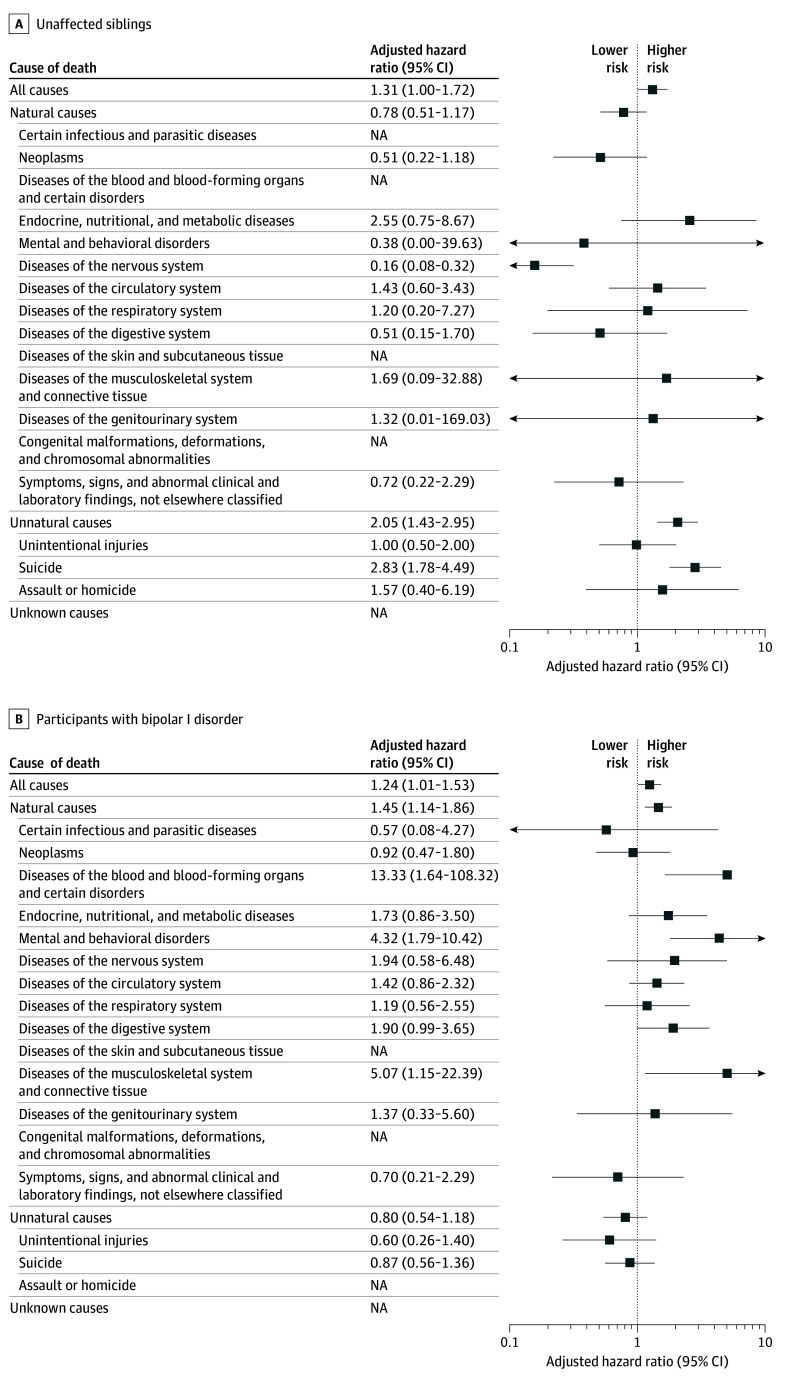
Forest Plot of All-Cause and Cause-Specific Mortality in Bipolar II Disorder vs Alternative Comparators Hazard ratios were adjusted for sex, birth year, income quartile, urbanization level, psychiatric health care use, and Charlson Comorbidity Index. NA indicates not available.

In the sibling cohort, 5063 participants with BD-II had 8002 unaffected siblings (baseline characteristics detailed in eTable 11 in Supplement 1). Relative to their siblings, participants with BD-II were more often female, had lower personal income, and carried greater medical and psychiatric comorbidity burdens. During follow-up, 174 participants with BD-II and 158 siblings died, corresponding to crude mortality rates of 1.6 and 0.9 per 1000 person-years, respectively. Adjusted risks (model 2) aligned with the population-matched analysis for all-cause mortality (AHR, 1.31; 95% CI, 1.00-1.72) and for deaths from unnatural causes (AHR, 2.05; 95% CI, 1.43-2.95), whereas natural-cause mortality did not differ (AHR, 0.78; 95% CI, 0.51-1.17) (Figure 2A; eTable 12 in Supplement 1). By specific causes, BD-II was associated with a lower risk of neurologic-related death (AHR, 0.16; 95% CI, 0.08-0.32) and a higher risk of suicide (AHR, 2.83; 95% CI, 1.78-4.49) than among unaffected siblings; several other cause-specific estimates were imprecise owing to limited power.

Characteristics of the BD-I cohort (n = 179 674) are summarized in eTable 11 in Supplement 1. Compared with the BD-I cohort, those with BD-II showed broadly similar distributions of sex, age, personal income, and medical and psychiatric comorbidity. Overall, 29 177 participants with BD-I died during follow-up (crude mortality rate, 14.8 per 1000 person-years). Compared with the BD-I cohort, participants with BD-II had higher adjusted risks (model 2) of all-cause mortality (AHR, 1.24; 95% CI, 1.01-1.53) and natural-cause mortality (AHR, 1.45; 95% CI, 1.14-1.86) but not unnatural-cause mortality (AHR, 0.80; 95% CI, 0.54-1.18) (Figure 2B; eTable 13 in Supplement 1). For specific causes, the BD-II cohort had elevated mortality risks for diseases of the blood and blood-forming organs and certain disorders, mental and behavioral disorders, and diseases of the musculoskeletal system and connective tissue, whereas suicide risk did not differ (AHR, 0.87; 95% CI, 0.56-1.36).

## Discussion

In this population-based cohort, individuals with BD-II had a 1.6-fold higher all-cause mortality than matched controls, with excess risk observed for both natural and, more prominently, unnatural causes, driven by unintentional injury, suicide, and assault or homicide. These patterns were broadly consistent across sex and age strata and were little changed when each of 7 psychiatric comorbidity groups was entered sequentially. In within-family analyses comparing participants with BD-II with their unaffected siblings, the elevations in all-cause and unnatural-cause mortality persisted, whereas the association with natural-cause mortality was no longer evident. In cross-subtype analyses, BD-II was associated with higher all-cause and natural-cause mortality than BD-I, with no difference in unnatural-cause mortality.

Our study addresses a key evidence gap by focusing specifically on BD-II and quantifying its mortality burden. Individuals with BD-II had higher all-cause, natural-cause, and unnatural-cause mortality than matched controls. Consistent with prior reports of substantial medical comorbidity in BD-II,^1,10^ the excess natural-cause mortality spanned multiple *ICD-9-CM* and *ICD-10-CM* chapters: mental and behavioral disorders; circulatory, respiratory, and digestive diseases; diseases of the skin and subcutaneous tissue; and symptoms, signs, and abnormal clinical and laboratory findings, not elsewhere classified. Several biological pathways may contribute to this pattern, including low-grade systemic inflammation,^32^ excess oxidative stress,^33^ and hypothalamic-pituitary-adrenal axis dysregulation^34^; moreover, BD has been associated with accelerated biological aging, reflected in reduced telomere length relative to individuals without the disorder.^35^ Additionally, atypical depressive symptoms common in BD-II, including hyperphagia, marked fatigue, and resultant low physical activity, may increase the risk of obesity and consequences, such as metabolic dysfunction–associated fatty liver disease, cardiovascular disease, and diabetes.^36^ Taken together, our findings align with contemporary models that conceptualize BD as a somatoprogressive illness^37^ and reinforce the need for integrated psychiatric and medical care. In addition, unnatural-cause mortality was also elevated, particularly for suicide, aligning with longstanding evidence that BD-II carries an especially high burden of suicidal behavior.^13,38^

Although overall patterns were broadly similar across sex, age, and psychiatric comorbidity strata, several nuances warrant attention. First, the relative risk of unnatural-cause death was higher in females with BD-II than in males vs their respective controls. This finding likely reflects the lower baseline rate of unnatural deaths among female controls, which magnifies the HR in females, consistent with prior literature on unintentional injuries and suicide.^39,40^ Second, adults with BD-II showed patterns similar to the overall results, whereas adolescents displayed a higher all-cause death risk, plausibly because natural-cause deaths are rare at younger ages and all-cause mortality is therefore dominated by unnatural causes. Third, results stratified by each of the 7 psychiatric-comorbidity groups paralleled the main analysis, and sequential adjustment produced only modest change, suggesting that the association between BD-II and mortality is not fully explained by these comorbidities.

We incorporated 2 complementary comparison designs to address distinct inferential aims: a within-family sibling comparison to reduce confounding by shared familial factors (genetic liability and early-life environment) and a cross-subtype comparison with a BD-I cohort. Both approaches indicated higher all-cause mortality in BD-II relative to its comparator, underscoring the mortality burden associated with BD-II. However, several comparator-specific differences warrant emphasis. For natural-cause mortality, patterns diverged across designs: in the sibling comparison, risk did not differ overall, whereas relative to BD-I, natural-cause mortality was unexpectedly higher in BD-II. Taken together, these findings suggest that part of the natural-cause excess observed in population-based analyses may reflect familial confounding, whereas the remainder appears attributable to subtype-specific mechanisms. Notably, despite comparable baseline medical comorbidity between BD-II and BD-I, BD-II was associated with higher natural-cause mortality. This counterintuitive finding may reflect differences in clinical course: longer depressive burden and more frequent delays in recognition and treatment of BD-II could reduce engagement with physical health care and allow greater accumulation of medical risk.^3,4^ The longstanding characterization of BD-II as milder may further contribute to lower clinical vigilance for physical health monitoring in these patients.^1^ By contrast, unnatural-cause mortality showed the opposite pattern. Relative to unaffected siblings, BD-II was associated with an elevated risk, driven particularly by suicide. In cross-subtype analyses, BD-II and BD-I did not differ for unintentional injuries or suicide. These findings indicate that the excess suicide risk in BD-II persists beyond shared familial factors. They also align with meta-analytic evidence showing no difference in suicide mortality between BD-II and BD-I,^14^ and our results extend this equivalence to unintentional injuries.

### Strengths and Limitations

This study has several strengths. Strengths include the large, population-based cohort and the availability of multiple comparator groups (matched controls, unaffected siblings, and participants with BD-I), enhancing both internal and external validity.

This study also has limitations. First, the NHIRD lacks standardized ratings of mood symptom severity, measures of episode frequency or number, and nondisease covariates known to influence mortality (eg, smoking, diet, and occupational exposures), precluding adjustment for these factors. Second, 2 sample characteristics warrant consideration: the low proportion of BD-II in the national population (<0.1%) and the relatively older mean age at first diagnosis (39.6 years). The former reflects our requirement for 2 or more psychiatrist-assigned *ICD-9-CM* or *ICD-10-CM* codes rather than screening tool–based ascertainment, likely enriching the cohort for clinically overt BD-II while excluding subthreshold cases.^41^ The latter reflects well-documented diagnostic delays of 5 to 10 years in BD-II rather than true age at onset,^1,42^ consistent with other register-based studies^42,43^ reporting first diagnosis ages of approximately 40 years. Third, the study was conducted in an East Asian population within Taiwan’s health care system; generalizability to settings with different medical practices or lifestyle patterns is uncertain, underscoring the need for replication in other jurisdictions.

## Conclusions

In this population-based cohort study integrating matched-population, within-family, and cross-subtype comparisons, BD-II was associated with consistently elevated all-cause mortality across sex, age, and psychiatric comorbidity strata. Cross-subtype analyses indicated higher all-cause and natural-cause mortality in BD-II than in BD-I. Excess unnatural-cause mortality, including suicide, was evident relative to matched controls and persisted in within-family analyses. Collectively, these findings suggest the need for strengthened surveillance, targeted prevention, and proactive comprehensive psychiatric care, with emphasis on early treatment of BD-II.

## Notes

Supplement 1. **eMethods. **Supplementary methods **eTable 1.** Diagnosis codes for causes of death **eTable 2. **Diagnosis codes for psychiatric disorders **eTable 3. **Psychiatric medications used for bipolar disorder in Taiwan **eTable 4. **Risk of all-cause and cause-specific mortality in participants with bipolar II disorder and matched controls, excluding participants with missing data **eTable 5. **Risk of all-cause and cause-specific mortality in participants with bipolar II disorder and matched controls, including bipolar II-to-I diagnostic converters **eTable 6.** Risk of all-cause and cause-specific mortality in participants with bipolar II disorder and matched controls, by sex (female) **eTable 7. **Risk of all-cause and cause-specific mortality in participants with bipolar II disorder and matched controls, by sex (male) **eTable 8. **Risk of all-cause and cause-specific mortality in participants with bipolar II disorder and matched controls, by age (adolescents) **eTable 9. **Risk of all-cause and cause-specific mortality in participants with bipolar II disorder and matched controls, by age (adults) **eTable 10. **Risk of all-cause and cause-specific mortality in participants with bipolar II disorder and matched controls, by age (older adults) **eTable 11.** Characteristics of all participants with bipolar II disorder (at least one sibling) and bipolar I disorder **eTable 12.** Risk of all-cause and cause-specific mortality in participants with bipolar II disorder and their unaffected siblings **eTable 13.** Risk of all-cause and cause-specific mortality in participants with bipolar II disorder and participants with bipolar I disorder **eFigure 1.** Flowchart of the selection process for the study **eFigure 2.** Kaplan-Meier survival curves for all-cause mortality in participants with bipolar II disorder (case) and matched controls (control)
eReferences

Supplement 2.
Data Sharing Statement
