# Supplementary material for: All-Cause and Cause-Specific Mortality in Patients With Bipolar II Disorder
Source: JAMA Netw Open. 2026 Apr 7;9(4):e265535. doi: 10.1001/jamanetworkopen.2026.5535 (PMC13058765; doi:10.1001/jamanetworkopen.2026.5535)
Supplement: Supplement 1. — eMethods. Supplementary methods eTable 1. Diagnosis codes for causes of death eTable 2. Diagnosis codes for psychiatric disorders eTable 3. Psychiatric medications used for bipolar disorder in Taiwan eTable 4. Risk of all-cause and cause-specific mortality in participants with bipolar II disorder and matched controls, excluding participants with missing data eTable 5. Risk of all-cause and cause-specific mortality in participants with bipolar II disorder and matched controls, including bipolar II-to-I diagnostic converters eTable 6. Risk of all-cause and cause-specific mortality in participants with bipolar II disorder and matched controls, by sex (female) eTable 7. Risk of all-cause and cause-specific mortality in participants with bipolar II disorder and matched controls, by sex (male) eTable 8. Risk of all-cause and cause-specific mortality in participants with bipolar II disorder and matched controls, by age (adolescents) eTable 9. Risk of all-cause and cause-specific mortality in participants with bipolar II disorder and matched controls, by age (adults) eTable 10. Risk of all-cause and cause-specific mortality in participants with bipolar II disorder and matched controls, by age (older adults) eTable 11. Characteristics of all participants with bipolar II disorder (at least one sibling) and bipolar I disorder eTable 12. Risk of all-cause and cause-specific mortality in participants with bipolar II disorder and their unaffected siblings eTable 13. Risk of all-cause and cause-specific mortality in participants with bipolar II disorder and participants with bipolar I disorder eFigure 1. Flowchart of the selection process for the study eFigure 2. Kaplan-Meier survival curves for all-cause mortality in participants with bipolar II disorder (case) and matched controls (control) eReferences [file jamanetwopen-e265535-s001.pdf]

Supplemental Online Content

Hsu C-W, Chen Y-CB. Lai EC-C. All-cause and cause-specific mortality in patients with bipolar II disorder. *JAMA Netw Open*. 2026;9(4):e265535. doi:10.1001/jamanetworkopen.2026.5535

eMethods. Supplementary methods
eTable 1. Diagnosis codes for causes of death
eTable 2. Diagnosis codes for psychiatric disorders
eTable 3. Psychiatric medications used for bipolar disorder in Taiwan
eTable 4. Risk of all-cause and cause-specific mortality in participants with bipolar II disorder and matched controls, excluding participants with missing data
eTable 5. Risk of all-cause and cause-specific mortality in participants with bipolar II disorder and matched controls, including bipolar II-to-I diagnostic converters
eTable 6. Risk of all-cause and cause-specific mortality in participants with bipolar II disorder and matched controls, by sex (female)
eTable 7. Risk of all-cause and cause-specific mortality in participants with bipolar II disorder and matched controls, by sex (male)
eTable 8. Risk of all-cause and cause-specific mortality in participants with bipolar II disorder and matched controls, by age (adolescents)
eTable 9. Risk of all-cause and cause-specific mortality in participants with bipolar II disorder and matched controls, by age (adults)
eTable 10. Risk of all-cause and cause-specific mortality in participants with bipolar II disorder and matched controls, by age (older adults)
eTable 11. Characteristics of all participants with bipolar II disorder (at least one sibling) and bipolar I disorder
eTable 12. Risk of all-cause and cause-specific mortality in participants with bipolar II disorder and their unaffected siblings
eTable 13. Risk of all-cause and cause-specific mortality in participants with bipolar II disorder and participants with bipolar I disorder
eFigure 1. Flowchart of the selection process for the study
eFigure 2. Kaplan-Meier survival curves for all-cause mortality in participants with bipolar II disorder (case) and matched controls (control)
eReferences

This supplemental material has been provided by the authors to give readers additional information about their work.

© 2026 Hsu C-W et al. *JAMA Network Open*.

eMethods. Supplementary methods

Study Cohorts

The Taiwan National Health Insurance Research Database (NHIRD) is a nationwide claims repository established in 1995 that captures healthcare utilization for over 99% of residents. It provides de-identified, linkable beneficiary-level records encompassing demographics (birth date, sex, income-indexed premium, residential region, and insured–dependent kinship) and complete outpatient/inpatient encounters, including International Classification of Diseases, Clinical Modification (ICD-CM)–coded diagnoses (ICD-9-CM through 2015; ICD-10-CM from 2016 onward), procedures, and dispensed prescriptions. Each beneficiary is assigned a unique encrypted identifier enabling secure deterministic linkage across NHIRD datasets and to external sources, notably Taiwan's National Death Registry, which supplies exact dates and certified causes of death. The database's scope and near-universal coverage—together with validation studies reporting high diagnostic accuracy (positive predictive values around 90% for many major conditions)—underscore its integrity and reliability. Consequently, the NHIRD serves as a premier platform for population-based epidemiology and generation of real-world evidence.[1,2]

For this analysis, we used NHIRD claims from 2000–2022. We constructed the BD-II cohort as follows: (1) To increase confidence in the veracity of diagnosis, we first identified individuals who received ≥2 clinical diagnoses of BD-II (ICD-9-CM 296.89; ICD-10-CM F31.81) from a board-certified psychiatrist between January 1, 2001, and December 31, 2021; (2) we excluded patients with unspecified sex or date of birth to ensure complete demographic data; (3) we excluded patients with any diagnosis of BD-I recorded throughout the entire database period (2000–2022), regardless of its temporal relationship to the index BD-II diagnosis (ICD-9-CM 296.0, 296.1, 296.4–296.7; ICD-10-CM F30.1–F30.4, F30.9, F31.0–F31.7, F31.89); and (4) we restricted the analytic sample to participants aged ≥12 years at the index BD-II diagnosis to enhance diagnostic accuracy.[3] To contextualize mortality risk, we formed a population-matched control cohort: for each individual with BD-II, four individuals from the general NHIRD population with no record of BD-II across their entire claims history were randomly selected and individually matched on sex and birth date (±6 months). We additionally established two comparison cohorts to examine mortality in greater depth. First, an unaffected sibling cohort: among BD-II patients with at least one sibling identifiable in the database (defined as beneficiaries sharing ≥1 biological parent), we included each index case and all biologically related brothers or sisters with no diagnosis of any bipolar disorder (BD-II and BD-I), enabling environment matched within-family contrasts. Second, a BD-I cohort: using criteria analogous to those for BD-II, we identified individuals with BD-I during 2001–2021. Comparison of the BD-II and BD-I cohorts allowed evaluation of subtype differences in mortality risk. The overall study workflow, including eligibility criteria and cohort construction, is shown in **eFigure 1**.

We assigned cohort-specific index dates as follows: (1) Population-matched controls: the index date

© 2026 Hsu C-W et al. *JAMA Network Open*.

for each matched set was the patient's first BD-II diagnosis; the same calendar date was assigned to all matched controls. (2) Unaffected siblings: January 1, 2001 served as the index date for both BD-II index patients and their unaffected siblings (the earliest date with comprehensive NHIRD coverage in this study). (3) BD-I cohort: the index date for BD-II patients was their first BD-II diagnosis, whereas the index date for BD-I patients was their first BD-I diagnosis. In all cohorts, follow-up extended from the assigned index date until death or December 31, 2022, whichever occurred first.

Outcomes and Covariates

The primary outcome was all-cause mortality. Secondary outcomes were cause-specific mortality categorized by ICD chapter into (1) natural causes, comprising all deaths other than those coded under "external causes of morbidity and mortality"; and (2) unnatural causes, comprising all external-cause codes. Unnatural deaths were further subtyped as unintentional injury, suicide, or assault or homicide. Deaths with missing or non-classifiable ICD codes were categorized as of unknown cause.

Baseline characteristics included (1) sex, (2) age at cohort entry, (3) individual income level, (4) residential urbanization, (5) psychiatric healthcare utilization, (6) medical comorbidities, (7) psychiatric comorbidities, and (8) psychiatric medication use. Income was stratified into quartiles relative to the matched-control population—highest (>75th percentile), upper-middle (50th–75th), lower-middle (25th–50th), and lowest (≤25th)—to provide a database-wide proxy for socioeconomic status. Residential urbanization, an established proxy for healthcare accessibility in Taiwan, was classified on a four-tier scale (levels 1–4, most to least urbanized) based on local population density, age structure, share of agricultural employment, prevalence of post-secondary education, and availability of medical providers.[4] Psychiatric healthcare utilization was characterized by the number of psychiatric hospitalizations (0, 1, or ≥2) and the number of psychiatric outpatient visits (0–6 [approximately bimonthly or less], 7–13 [approximately monthly], or ≥14 [more than monthly]). Medical comorbidity burden was summarized using the Charlson Comorbidity Index (CCI), a validated measure that integrates 19 chronic conditions—including dementia; cerebrovascular and peripheral vascular disease; myocardial infarction; congestive heart failure; chronic pulmonary disease; diabetes (with and without complications); renal disease; peptic ulcer disease; liver disease (mild and severe); rheumatic disease; malignancy (non-metastatic and metastatic); paraplegia; and acquired immunodeficiency syndrome—to estimate overall morbidity and long-term mortality risk.[5] Psychiatric comorbidities were classified into seven diagnostic groups: neurodevelopmental disorders (intellectual disability, autism spectrum disorder, attention-deficit/hyperactivity disorder, Tourette syndrome, and chronic tic disorder),[6] anxiety disorders,[7] obsessive-compulsive disorder,[8] post-traumatic stress disorder,[9] eating disorders,[10] substance use disorders,[11] and personality disorders.[12] These groups were selected because prior studies indicate that each is independently associated with elevated mortality risk. Psychiatric medication use was assessed for three classes: antidepressants, antipsychotics, and mood

© 2026 Hsu C-W et al. *JAMA Network Open*.

stabilizers; use of each class was defined as having at least one prescription with a duration of ≥14 days. Healthcare utilization, medical comorbidities, psychiatric comorbidities, and psychiatric medication use were ascertained from the one-year period up to and including the index date. Comprehensive ICD code lists for cause-of-death categories and psychiatric disorders are provided in **eTable 1–2**, and the list of psychiatric medications used for bipolar disorder in Taiwan is provided in **eTable 3**.

Statistical Analyses

Baseline characteristics were summarized as counts (percentages) for categorical variables and as means (standard deviations) for continuous variables. We plotted Kaplan–Meier curves to visualize survival and compared groups using the log-rank test. We fitted Cox proportional hazards models to estimate hazard ratios (HRs) with 95% confidence intervals (CIs), using time since cohort entry as the underlying time scale. Analyses were first conducted for all-cause mortality and then repeated for each cause-specific outcome. Model 1 reported crude HRs; Model 2 reported adjusted HRs, additionally adjusting for sex, birth year, income quartile, urbanization level, psychiatric healthcare utilization (number of psychiatric hospitalizations and psychiatric outpatient visits), and CCI. Psychiatric medication use was reported descriptively but not included as a covariate. Because these medications are typically prescribed in relation to the psychiatric diagnosis, they may more appropriately be considered mediators rather than confounders, and their inclusion could introduce overadjustment bias.[13,14] Missing covariate values were coded as “unknown” and retained as explicit categories in the models.

We conducted two sensitivity analyses, three subgroup analyses, and two comparator analyses. Sensitivity analyses repeated the primary Cox models (Models 1–2): the first was restricted to a complete-case sample after excluding participants with missing covariate data; the second reincluded 2164 patients who initially received a BD-II diagnosis but subsequently received a BD-I diagnosis during the database period (**eFigure 1**), to assess the robustness of findings to diagnostic conversion. Subgroup analyses stratified by sex (female, male), age (adolescents 12–17 years, adults 18–64 years, older adults ≥65 years), and psychiatric comorbidities. To assess the contribution of psychiatric comorbidities to the association between BD-II and mortality, we re-estimated Model 2 while sequentially adding each comorbidity group as an adjustment variable. Comparator analyses contrasted (1) BD-II patients with ≥1 sibling vs their unaffected siblings and (2) BD-II vs BD-I patients. The same modeling framework (Models 1–2) was applied; for the sibling comparison, Model 2 additionally adjusted for sibling birth order and used cluster-robust (sandwich) standard errors to account for within-family correlation.[15] All analyses were performed in SAS 9.4 (SAS Institute Inc., Cary, NC, USA). A two-sided $P < 0.05$ was considered statistically significant for all tests; for Cox models, HRs were considered statistically significant when the 95% CI did not include 1.00. Statistical analysis was performed from June to August 2025.

© 2026 Hsu C-W et al. *JAMA Network Open*.

eTable 1. Diagnosis codes for causes of death

Specific cause of death	ICD-9 codes	ICD-10 codes
**Natural causes**	001–799	A–R
Certain infectious and parasitic diseases	001–139	A00–B99
Neoplasms	140–239	C00–D49
Diseases of the blood and blood-forming organs and certain disorders (involving the immune mechanism)	279–289	D50–D89
Endocrine, nutritional, and metabolic diseases	240–278	E00–E90
Mental and behavioral disorders	290–319	F00–F99
Diseases of the nervous system	320–359	G00–G99
Diseases of the eye and adnexa	360–379	H00–H59
Diseases of the ear and mastoid process	380–389	H60–H95
Diseases of the circulatory system	390–459	I00–I99
Diseases of the respiratory system	460–519	J00–J99
Diseases of the digestive system	520–579	K00–K93
Diseases of the skin and subcutaneous tissue	680–709	L00–L99
Diseases of the musculoskeletal system and connective tissue	710–739	M00–M99
Diseases of the genitourinary system	580–629	N00–N99
Pregnancy, childbirth, and the puerperium	630–679	O00–O99
Certain conditions originating in the perinatal period	760–779	P00–P96
Congenital malformations, deformations and chromosomal abnormalities	740–759	Q00–Q99
Symptoms, signs and abnormal clinical and laboratory findings, not elsewhere classified	780–799	R00–R99
**Unnatural causes (external causes of morbidity and mortality)**	E800–E969, E980–E989	V01–Y98
Unintentional injuries	E800–E949	V01–X59, Y40–Y86, Y88, Y89
Suicide	E950–E959, E980–E989	X60–X84, Y10–Y34
Assault or homicide	E960–E969	X85–X99, Y00–Y09
**Unknown causes**	Missing or non-classifiable	Missing or non-classifiable

Abbreviation: ICD, International Classification of Diseases

© 2026 Hsu C-W et al. *JAMA Network Open*.

eTable 2. Diagnosis codes for psychiatric disorders

	ICD-9 codes	ICD-10 codes
**Bipolar II disorder**	296.89	F31.81
**Bipolar I disorder**	296.0, 296.1, 296.4–296.7	F30.1–F30.4, F30.9, F31.0–F31.7, F31.89
**Neurodevelopmental disorders**		
(Intellectual disability)	317–319	F70–F73, F78, F79
(Autism spectrum disorders)	299	F84
(Attention-deficit/hyperactivity disorder)	314	F90
(Tourette syndrome and tic disorder)	307.2	F95
**Anxiety disorders**	300.0, 300.2, 309.21	F40, F41, F93.0
**Obsessive-compulsive disorder**	300.3	F42
**Post-traumatic stress disorder**	309.81	F43.1
**Eating disorders**	307.1, 307.5	F50
**Substance use disorders**	303, 304, 305.0, 305.2, 305.3, 305.4, 305.5, 305.6, 305.7, 305.8, 305.9	F10–F16, F18, F19
**Personality disorders**	301	F60

Abbreviation: ICD, International Classification of Diseases

© 2026 Hsu C-W et al. *JAMA Network Open*.

eTable 3. Psychiatric medications used for bipolar disorder in Taiwan

Medication class	Included drugs
**Antidepressants**	agomelatine, amitriptyline, amoxapine, bupropion, citalopram, clomipramine, desipramine, desvenlafaxine, dosulepin, doxepin, duloxetine, escitalopram, fluoxetine, fluvoxamine, imipramine, isocarboxazid, levomilnacipran, maprotiline, mianserin, milnacipran, mirtazapine, moclobemide, nortriptyline, paroxetine, phenelzine, protriptyline, sertraline, tranylcypromine, trazodone, trimipramine, venlafaxine, vortioxetine
**Antipsychotics**	amisulpride, aripiprazole, brexpiprazole, chlorpromazine, chlorprothixene, clopenthixol, clozapine, flupentixol, fluphenazine, haloperidol, loxapine, lurasidone, olanzapine, paliperidone, perphenazine, pipotiazine, quetiapine, risperidone, sulpiride, thioridazine, tiotixene, trifluoperazine, ziprasidone, zotepine, zuclopenthixol
**Mood stabilizers**	carbamazepine, lamotrigine, lithium, topiramate, valproic acid

© 2026 Hsu C-W et al. *JAMA Network Open*.

eTable 4. Risk of all-cause and cause-specific mortality in participants with bipolar II disorder and matched controls, excluding participants with missing data

Characteristics	Case, event (n = 10,695)	Control, event (n = 39,976)	Model 1 (Crude hazard ratio)	Model 2 (Adjusted hazard ratio)
**All-cause**	994 (9.3)	1481 (3.7)	2.66 (2.46–2.88)	1.69 (1.52–1.87)
**Natural causes**	716 (6.7)	1314 (3.3)	2.16 (1.98–2.37)	1.41 (1.26–1.58)
Certain infectious and parasitic diseases	18 (0.2)	29 (0.1)	2.45 (1.36–4.42)	1.20 (0.57–2.55)
Neoplasms	160 (1.5)	456 (1.1)	1.39 (1.16–1.67)	1.12 (0.90–1.39)
Diseases of the blood and blood-forming organs and certain disorders	4 (<0.1)	4 (<0.1)	3.89 (0.97–15.56)	2.52 (0.51–12.4)
Endocrine, nutritional, and metabolic diseases	63 (0.6)	108 (0.3)	2.32 (1.70–3.17)	1.13 (0.76–1.67)
Mental and behavioral disorders	17 (0.2)	14 (<0.1)	4.85 (2.39–9.83)	2.49 (0.98–6.29)
Diseases of the nervous system	20 (0.2)	30 (0.1)	2.67 (1.52–4.71)	1.69 (0.81–3.53)
Diseases of the eye and adnexa	0 (0.0)	0 (0.0)	–	–
Diseases of the ear and mastoid process	0 (0.0)	0 (0.0)	–	–
Diseases of the circulatory system	176 (1.6)	334 (0.8)	2.10 (1.75–2.52)	1.44 (1.14–1.81)
Diseases of the respiratory system	95 (0.9)	127 (0.3)	2.98 (2.28–3.89)	1.69 (1.19–2.40)
Diseases of the digestive system	76 (0.7)	90 (0.2)	3.34 (2.46–4.53)	2.07 (1.42–3.03)
Diseases of the skin and subcutaneous tissue	3 (<0.1)	0 (0.0)	–	–
Diseases of the musculoskeletal system and connective tissue	8 (0.1)	8 (<0.1)	3.92 (1.47–10.46)	1.96 (0.59–6.55)
Diseases of the genitourinary system	25 (0.2)	63 (0.2)	1.57 (0.99–2.50)	1.01 (0.58–1.76)
Pregnancy, childbirth, and the puerperium	0 (0.0)	2 (<0.1)	–	–
Certain conditions originating in the perinatal period	0 (0.0)	0 (0.0)	–	–
Congenital malformations, deformations and chromosomal abnormalities	1 (<0.1)	2 (<0.1)	1.99 (0.18–21.91)	2.75 (0.24–32.11)
Symptoms, signs and abnormal clinical and laboratory findings, not elsewhere classified	50 (0.5)	47 (0.1)	4.22 (2.83–6.28)	2.44 (1.44–4.14)
**Unnatural causes**	271 (2.5)	142 (0.4)	7.48 (6.11–9.17)	4.61 (3.60–5.90)
Unintentional injuries	78 (0.7)	69 (0.2)	4.45 (3.22–6.15)	2.99 (1.99–4.48)
Suicide	189 (1.8)	70 (0.2)	10.56 (8.03–13.90)	6.12 (4.42–8.47)
Assault or homicide	4 (<0.1)	3 (<0.1)	5.14 (1.15–22.95)	6.36 (1.25–32.43)
**Unknown causes**	7 (0.1)	25 (0.1)	1.12 (0.49–2.60)	0.52 (0.17–1.60)

[1] Event was expressed as N (percentage).

[2] Model 2 adjusted for all variables (sex, birth year, income level, urbanization level, psychiatric healthcare utilization, and Charlson Comorbidity Index).

© 2026 Hsu C-W et al. *JAMA Network Open*.

eTable 5. Risk of all-cause and cause-specific mortality in participants with bipolar II disorder and matched controls, including bipolar II-to-I diagnostic converters

Characteristics	Case, event (n = 13,591)	Control, event (n = 54,364)	Model 1 (Crude hazard ratio)	Model 2 (Adjusted hazard ratio)
**All-cause**	1312 (9.7)	2201 (4.0)	2.51 (2.34–2.68)	1.65 (1.51–1.80)
**Natural causes**	928 (6.8)	1965 (3.6)	1.99 (1.84–2.15)	1.37 (1.24–1.51)
Certain infectious and parasitic diseases	22 (0.2)	43 (0.1)	2.14 (1.28–3.58)	1.13 (0.58–2.21)
Neoplasms	208 (1.5)	677 (1.2)	1.29 (1.11–1.51)	1.05 (0.87–1.27)
Diseases of the blood and blood-forming organs and certain disorders	4 (<0.1)	6 (<0.1)	2.77 (0.78–9.80)	1.84 (0.42–8.04)
Endocrine, nutritional, and metabolic diseases	79 (0.6)	161 (0.3)	2.07 (1.58–2.71)	1.10 (0.78–1.56)
Mental and behavioral disorders	28 (0.2)	28 (0.1)	4.22 (2.50–7.13)	2.92 (1.47–5.79)
Diseases of the nervous system	28 (0.2)	41 (0.1)	2.89 (1.79–4.68)	1.85 (0.98–3.52)
Diseases of the eye and adnexa	0 (0.0)	0 (0.0)	–	–
Diseases of the ear and mastoid process	0 (0.0)	0 (0.0)	–	–
Diseases of the circulatory system	227 (1.7)	487 (0.9)	1.97 (1.68–2.30)	1.39 (1.13–1.71)
Diseases of the respiratory system	122 (0.9)	200 (0.4)	2.57 (2.05–3.22)	1.66 (1.23–2.23)
Diseases of the digestive system	88 (0.6)	124 (0.2)	2.97 (2.26–3.91)	1.86 (1.32–2.62)
Diseases of the skin and subcutaneous tissue	4 (<0.1)	3 (<0.1)	5.56 (1.24–24.83)	11.01 (1.96–61.70)
Diseases of the musculoskeletal system and connective tissue	12 (0.1)	11 (<0.1)	4.58 (2.02–10.38)	2.98 (1.13–7.87)
Diseases of the genitourinary system	41 (0.3)	94 (0.2)	1.84 (1.27–2.65)	1.37 (0.88–2.12)
Pregnancy, childbirth, and the puerperium	0 (0.0)	2 (<0.1)	–	–
Certain conditions originating in the perinatal period	0 (0.0)	0 (0.0)	–	–
Congenital malformations, deformations and chromosomal abnormalities	1 (<0.1)	2 (<0.1)	2.10 (0.19–23.18)	2.95 (0.25–34.71)
Symptoms, signs and abnormal clinical and laboratory findings, not elsewhere classified	64 (0.5)	86 (0.2)	3.13 (2.26–4.32)	1.79 (1.14–2.78)
**Unnatural causes**	375 (2.8)	207 (0.4)	7.55 (6.37–8.95)	4.64 (3.76–5.74)
Unintentional injuries	111 (0.8)	104 (0.2)	4.47 (3.42–5.84)	2.69 (1.89–3.81)
Suicide	260 (1.9)	100 (0.2)	10.81 (8.59–13.62)	6.60 (5.00–8.71)
Assault or homicide	4 (<0.1)	3 (<0.1)	5.46 (1.22–24.37)	6.96 (1.36–35.58)
**Unknown causes**	9 (0.1)	29 (0.1)	1.32 (0.63–2.79)	0.50 (0.17–1.42)

[1] Event was expressed as N (percentage).

[2] Model 2 adjusted for all variables (sex, birth year, income level, urbanization level, psychiatric healthcare utilization, and Charlson Comorbidity Index).

© 2026 Hsu C-W et al. *JAMA Network Open*.

eTable 6. Risk of all-cause and cause-specific mortality in participants with bipolar II disorder and matched controls, by sex (female)

Characteristics	Case, event (n = 7073)	Control, event (n = 28,292)	Model 1 (Crude hazard ratio)	Model 2 (Adjusted hazard ratio)
**All-cause**	474 (6.7)	785 (2.8)	2.51 (2.24–2.82)	1.58 (1.37–1.83)
**Natural causes**	335 (4.7)	711 (2.5)	1.96 (1.72–2.24)	1.31 (1.11–1.54)
Certain infectious and parasitic diseases	7 (0.1)	16 (0.1)	1.81 (0.75–4.41)	0.70 (0.23–2.17)
Neoplasms	81 (1.1)	235 (0.8)	1.44 (1.11–1.85)	1.02 (0.75–1.39)
Diseases of the blood and blood-forming organs and certain disorders	1 (<0.1)	2 (<0.1)	2.03 (0.18–22.41)	2.69 (0.22–33.16)
Endocrine, nutritional, and metabolic diseases	39 (0.6)	71 (0.3)	2.29 (1.55–3.38)	1.14 (0.70–1.85)
Mental and behavioral disorders	4 (0.1)	9 (<0.1)	1.87 (0.58–6.08)	0.77 (0.14–4.18)
Diseases of the nervous system	8 (0.1)	15 (0.1)	2.22 (0.94–5.23)	1.30 (0.43–3.93)
Diseases of the eye and adnexa	0 (0.0)	0 (0.0)	–	–
Diseases of the ear and mastoid process	0 (0.0)	0 (0.0)	–	–
Diseases of the circulatory system	83 (1.2)	176 (0.6)	1.97 (1.52–2.56)	1.48 (1.07–2.05)
Diseases of the respiratory system	43 (0.6)	62 (0.2)	2.89 (1.96–4.27)	1.61 (0.97–2.67)
Diseases of the digestive system	16 (0.2)	30 (0.1)	2.21 (1.21–4.06)	2.03 (1.01–4.10)
Diseases of the skin and subcutaneous tissue	2 (<0.1)	1 (<0.1)	8.25 (0.75–90.94)	73.42 (2.52–2135.75)
Diseases of the musculoskeletal system and connective tissue	4 (0.1)	4 (<0.1)	4.13 (1.03–16.51)	4.28 (0.94–19.55)
Diseases of the genitourinary system	21 (0.3)	59 (0.2)	1.49 (0.90–2.45)	1.07 (0.60–1.93)
Pregnancy, childbirth, and the puerperium	0 (0.0)	2 (<0.1)	–	–
Certain conditions originating in the perinatal period	0 (0.0)	0 (0.0)	–	–
Congenital malformations, deformations and chromosomal abnormalities	0 (0.0)	0 (0.0)	–	–
Symptoms, signs and abnormal clinical and laboratory findings, not elsewhere classified	26 (0.4)	29 (0.1)	3.72 (2.19–6.31)	2.33 (1.17–4.65)
**Unnatural causes**	134 (1.9)	60 (0.2)	9.19 (6.78–12.47)	5.41 (3.74–7.83)
Unintentional injuries	27 (0.4)	30 (0.1)	3.73 (2.22–6.28)	2.72 (1.42–5.21)
Suicide	103 (1.5)	29 (0.1)	14.58 (9.65–22.01)	7.49 (4.60–12.18)
Assault or homicide	4 (0.1)	1 (<0.1)	16.30 (1.82–145.82)	28.93 (3.13–266.95)
**Unknown causes**	5 (0.1)	14 (<0.1)	1.50 (0.54–4.16)	0.31 (0.08–1.28)

[1] Event was expressed as N (percentage).

[2] Model 2 adjusted for all variables (sex, birth year, income level, urbanization level, psychiatric healthcare utilization, and Charlson Comorbidity Index).

© 2026 Hsu C-W et al. *JAMA Network Open*.

eTable 7. Risk of all-cause and cause-specific mortality in participants with bipolar II disorder and matched controls, by sex (male)

Characteristics	Case, event (n = 4354)	Control, event (n = 17,416)	Model 1 (Crude hazard ratio)	Model 2 (Adjusted hazard ratio)
**All-cause**	615 (14.1)	1094 (6.3)	2.41 (2.18–2.66)	1.66 (1.46–1.88)
**Natural causes**	458 (10.5)	974 (5.6)	2.02 (1.81–2.26)	1.43 (1.24–1.64)
Certain infectious and parasitic diseases	12 (0.3)	23 (0.1)	2.22 (1.10–4.46)	1.49 (0.62–3.56)
Neoplasms	93 (2.1)	316 (1.8)	1.26 (1.00–1.59)	1.17 (0.89–1.54)
Diseases of the blood and blood-forming organs and certain disorders	3 (0.1)	3 (<0.1)	4.23 (0.85–20.95)	2.14 (0.32–14.29)
Endocrine, nutritional, and metabolic diseases	29 (0.7)	74 (0.4)	1.68 (1.09–2.58)	0.94 (0.55–1.62)
Mental and behavioral disorders	17 (0.4)	14 (0.1)	5.23 (2.58–10.61)	3.05 (1.20–7.77)
Diseases of the nervous system	14 (0.3)	23 (0.1)	2.65 (1.36–5.15)	1.80 (0.74–4.38)
Diseases of the eye and adnexa	0 (0.0)	0 (0.0)	–	–
Diseases of the ear and mastoid process	0 (0.0)	0 (0.0)	–	–
Diseases of the circulatory system	114 (2.6)	253 (1.5)	1.94 (1.55–2.41)	1.39 (1.05–1.85)
Diseases of the respiratory system	65 (1.5)	114 (0.7)	2.46 (1.81–3.33)	1.62 (1.08–2.43)
Diseases of the digestive system	61 (1.4)	76 (0.4)	3.42 (2.44–4.80)	2.00 (1.30–3.06)
Diseases of the skin and subcutaneous tissue	2 (<0.1)	1 (<0.1)	8.43 (0.76–92.98)	9.93 (0.76–130.17)
Diseases of the musculoskeletal system and connective tissue	6 (0.1)	7 (<0.1)	3.67 (1.23–10.93)	1.73 (0.42–7.16)
Diseases of the genitourinary system	15 (0.3)	25 (0.1)	2.58 (1.36–4.89)	2.03 (0.96–4.29)
Pregnancy, childbirth, and the puerperium	0 (0.0)	0 (0.0)	–	–
Certain conditions originating in the perinatal period	0 (0.0)	0 (0.0)	–	–
Congenital malformations, deformations and chromosomal abnormalities	1 (<0.1)	2 (<0.1)	2.14 (0.19–23.67)	2.87 (0.25–33.69)
Symptoms, signs and abnormal clinical and laboratory findings, not elsewhere classified	26 (0.6)	43 (0.2)	2.60 (1.60–4.23)	1.51 (0.78–2.94)
**Unnatural causes**	154 (3.5)	106 (0.6)	6.16 (4.81–7.89)	4.01 (2.95–5.44)
Unintentional injuries	54 (1.2)	55 (0.3)	4.17 (2.86–6.07)	2.88 (1.79–4.62)
Suicide	100 (2.3)	49 (0.3)	8.64 (6.14–12.16)	5.52 (3.66–8.33)
Assault or homicide	0 (0.0)	2 (<0.1)	–	–
**Unknown causes**	3 (0.1)	14 (0.1)	0.94 (0.27–3.26)	0.97 (0.23–4.05)

[1] Event was expressed as N (percentage).

[2] Model 2 adjusted for all variables (sex, birth year, income level, urbanization level, psychiatric healthcare utilization, and Charlson Comorbidity Index).

© 2026 Hsu C-W et al. *JAMA Network Open*.

eTable 8. Risk of all-cause and cause-specific mortality in participants with bipolar II disorder and matched controls, by age (adolescents)

Characteristics	Case, event (n = 566)	Control, event (n = 2264)	Model 1 (Crude hazard ratio)	Model 2 (Adjusted hazard ratio)
**All-cause**	6 (1.1)	7 (0.3)	3.44 (1.16–10.23)	3.49 (1.01–12.03)
**Natural causes**	0 (0.0)	2 (0.1)	–	–
Certain infectious and parasitic diseases	0 (0.0)	0 (0.0)	–	–
Neoplasms	0 (0.0)	0 (0.0)	–	–
Diseases of the blood and blood-forming organs and certain disorders	0 (0.0)	0 (0.0)	–	–
Endocrine, nutritional, and metabolic diseases	0 (0.0)	0 (0.0)	–	–
Mental and behavioral disorders	0 (0.0)	0 (0.0)	–	–
Diseases of the nervous system	0 (0.0)	0 (0.0)	–	–
Diseases of the eye and adnexa	0 (0.0)	0 (0.0)	–	–
Diseases of the ear and mastoid process	0 (0.0)	0 (0.0)	–	–
Diseases of the circulatory system	0 (0.0)	1 (<0.1)	–	–
Diseases of the respiratory system	0 (0.0)	0 (0.0)	–	–
Diseases of the digestive system	0 (0.0)	1 (<0.1)	–	–
Diseases of the skin and subcutaneous tissue	0 (0.0)	0 (0.0)	–	–
Diseases of the musculoskeletal system and connective tissue	0 (0.0)	0 (0.0)	–	–
Diseases of the genitourinary system	0 (0.0)	0 (0.0)	–	–
Pregnancy, childbirth, and the puerperium	0 (0.0)	0 (0.0)	–	–
Certain conditions originating in the perinatal period	0 (0.0)	0 (0.0)	–	–
Congenital malformations, deformations and chromosomal abnormalities	0 (0.0)	0 (0.0)	–	–
Symptoms, signs and abnormal clinical and laboratory findings, not elsewhere classified	0 (0.0)	0 (0.0)	–	–
**Unnatural causes**	6 (1.1)	5 (0.2)	4.82 (1.47–15.78)	4.90 (1.30–18.52)
Unintentional injuries	1 (0.2)	2 (0.1)	2.01 (0.18–22.15)	3.44 (0.31–38.44)
Suicide	5 (0.9)	3 (0.1)	6.69 (1.60–27.98)	6.14 (1.21–31.00)
Assault or homicide	0 (0.0)	0 (0.0)	–	–
**Unknown causes**	0 (0.0)	0 (0.0)	–	–

[1] Event was expressed as N (percentage).

[2] Model 2 adjusted for all variables (sex, birth year, income level, urbanization level, psychiatric healthcare utilization, and Charlson Comorbidity Index).

© 2026 Hsu C-W et al. *JAMA Network Open*.

eTable 9. Risk of all-cause and cause-specific mortality in participants with bipolar II disorder and matched controls, by age (adults)

Characteristics	Case, event (n = 9847)	Control, event (n = 39,388)	Model 1 (Crude hazard ratio)	Model 2 (Adjusted hazard ratio)
**All-cause**	716 (7.3)	923 (2.3)	3.26 (2.95–3.59)	1.95 (1.72–2.21)
**Natural causes**	445 (4.5)	784 (2.0)	2.39 (2.13–2.69)	1.45 (1.25–1.68)
Certain infectious and parasitic diseases	12 (0.1)	14 (<0.1)	3.60 (1.67–7.79)	1.69 (0.61–4.67)
Neoplasms	103 (1.0)	339 (0.9)	1.28 (1.03–1.60)	1.05 (0.80–1.38)
Diseases of the blood and blood-forming organs and certain disorders	4 (<0.1)	2 (<0.1)	8.28 (1.52–45.23)	5.34 (0.81–35.14)
Endocrine, nutritional, and metabolic diseases	42 (0.4)	61 (0.2)	2.90 (1.96–4.30)	1.17 (0.70–1.96)
Mental and behavioral disorders	15 (0.2)	5 (<0.1)	12.62 (4.59–34.72)	7.30 (2.18-24.50)
Diseases of the nervous system	14 (0.1)	20 (0.1)	2.96 (1.49–5.86)	1.36 (0.53–3.49)
Diseases of the eye and adnexa	0 (0.0)	0 (0.0)	–	–
Diseases of the ear and mastoid process	0 (0.0)	0 (0.0)	–	–
Diseases of the circulatory system	100 (1.0)	162 (0.4)	2.61 (2.03–3.35)	1.62 (1.18–2.23)
Diseases of the respiratory system	42 (0.4)	46 (0.1)	3.86 (2.54–5.87)	1.99 (1.14–3.47)
Diseases of the digestive system	62 (0.6)	68 (0.2)	3.82 (2.71–5.39)	2.13 (1.39–3.27)
Diseases of the skin and subcutaneous tissue	0 (0.0)	1 (<0.1)	–	–
Diseases of the musculoskeletal system and connective tissue	4 (<0.1)	7 (<0.1)	2.40 (0.70–8.21)	1.99 (0.47–8.54)
Diseases of the genitourinary system	13 (0.1)	29 (0.1)	1.90 (0.99–3.65)	1.05 (0.47–2.38)
Pregnancy, childbirth, and the puerperium	0 (0.0)	2 (<0.1)	–	–
Certain conditions originating in the perinatal period	0 (0.0)	0 (0.0)	–	–
Congenital malformations, deformations and chromosomal abnormalities	1 (<0.1)	2 (<0.1)	2.10 (0.19–23.18)	3.04 (0.26–36.11)
Symptoms, signs and abnormal clinical and laboratory findings, not elsewhere classified	33 (0.3)	26 (0.1)	5.31 (3.18–8.89)	2.06 (1.02–4.18)
**Unnatural causes**	265 (2.7)	127 (0.3)	8.67 (7.02–10.71)	5.21 (4.03–6.74)
Unintentional injuries	71 (0.7)	59 (0.1)	5.03 (3.56–7.10)	3.13 (2.03–4.82)
Suicide	190 (1.9)	65 (0.2)	12.11 (9.14–16.05)	7.04 (5.04–9.83)
Assault or homicide	4 (<0.1)	3 (<0.1)	5.46 (1.22–24.38)	6.54 (1.29–33.30)
**Unknown causes**	6 (0.1)	12 (<0.1)	2.13 (0.80–5.66)	0.94 (0.25–3.50)

[1] Event was expressed as N (percentage).

[2] Model 2 adjusted for all variables (sex, birth year, income level, urbanization level, psychiatric healthcare utilization, and Charlson Comorbidity Index).

© 2026 Hsu C-W et al. *JAMA Network Open*.

eTable 10. Risk of all-cause and cause-specific mortality in participants with bipolar II disorder and matched controls, by age (older adults)

Characteristics	Case, event (n = 1014)	Control, event (n = 4056)	Model 1 (Crude hazard ratio)	Model 2 (Adjusted hazard ratio)
**All-cause**	367 (36.2)	949 (23.4)	1.83 (1.62–2.06)	1.31 (1.13–1.51)
**Natural causes**	348 (34.3)	899 (22.2)	1.83 (1.62–2.07)	1.33 (1.14–1.55)
Certain infectious and parasitic diseases	7 (0.7)	25 (0.6)	1.27 (0.55–2.94)	0.59 (0.20–1.72)
Neoplasms	71 (7.0)	212 (5.2)	1.54 (1.17–2.01)	1.14 (0.83–1.57)
Diseases of the blood and blood-forming organs and certain disorders	0 (0.0)	3 (0.1)	–	–
Endocrine, nutritional, and metabolic diseases	26 (2.6)	84 (2.1)	1.44 (0.93–2.24)	0.93 (0.55–1.57)
Mental and behavioral disorders	6 (0.6)	18 (0.4)	1.66 (0.66–4.20)	0.83 (0.24–2.82)
Diseases of the nervous system	8 (0.8)	18 (0.4)	2.18 (0.95–5.03)	2.05 (0.75–5.62)
Diseases of the eye and adnexa	0 (0.0)	0 (0.0)	–	–
Diseases of the ear and mastoid process	0 (0.0)	0 (0.0)	–	–
Diseases of the circulatory system	97 (9.6)	266 (6.6)	1.75 (1.39–2.21)	1.33 (0.998–1.76)
Diseases of the respiratory system	66 (6.5)	130 (3.2)	2.46 (1.83–3.32)	1.51 (1.03–2.21)
Diseases of the digestive system	15 (1.5)	37 (0.9)	1.87 (1.02–3.41)	1.82 (0.88–3.77)
Diseases of the skin and subcutaneous tissue	4 (0.4)	1 (<0.1)	17.77 (1.98–159.18)	29.24 (2.56–334.20)
Diseases of the musculoskeletal system and connective tissue	6 (0.6)	4 (0.1)	6.84 (1.93–24.30)	3.09 (0.70–13.69)
Diseases of the genitourinary system	23 (2.3)	55 (1.4)	1.99 (1.22–3.24)	1.54 (0.89–2.69)
Pregnancy, childbirth, and the puerperium	0 (0.0)	0 (0.0)	–	–
Certain conditions originating in the perinatal period	0 (0.0)	0 (0.0)	–	–
Congenital malformations, deformations and chromosomal abnormalities	0 (0.0)	0 (0.0)	–	–
Symptoms, signs and abnormal clinical and laboratory findings, not elsewhere classified	19 (1.9)	46 (1.1)	2.01 (1.17–3.43)	1.90 (1.01–3.59)
**Unnatural causes**	17 (1.7)	34 (0.8)	2.23 (1.25–4.00)	1.38 (0.63–2.99)
Unintentional injuries	9 (0.9)	24 (0.6)	1.69 (0.78–3.64)	1.83 (0.72–4.65)
Suicide	8 (0.8)	10 (0.2)	3.51 (1.38–8.90)	0.88 (0.23–3.41)
Assault or homicide	0 (0.0)	0 (0.0)	–	–
**Unknown causes**	2 (0.2)	16 (0.4)	0.63 (0.14–2.74)	0.21 (0.03–1.26)

[1] Event was expressed as N (percentage).

[2] Model 2 adjusted for all variables (sex, birth year, income level, urbanization level, psychiatric healthcare utilization, and Charlson Comorbidity Index).

© 2026 Hsu C-W et al. *JAMA Network Open*.

eTable 11. Characteristics of all participants with bipolar II disorder (at least one sibling) and bipolar I disorder

Characteristics	Unaffected sibling cohort		Bipolar I disorder cohort	
	Case (bipolar II disorder) (n = 5063)	Control (their sibling) (n = 8002)	Case (bipolar II disorder) (n = 11,427)	Control (bipolar I disorder) (n = 179,674)
**Basic information**				
Age, year	–	–	39.6 ± 16.6	40.9 ± 16.4
Sex, female / male	3109 (61.4) / 1954 (38.6)	3945 (49.3) / 4057 (50.7)	7073 (61.9) / 4354 (38.1)	103,691 (57.7) / 75,983 (42.3)
**Personal income level**				
>25th (highest)	764 (15.1)	1674 (20.9)	1845 (16.1)	27,003 (15.0)
25–50th	1208 (23.9)	1894 (23.7)	2208 (19.3)	38,472 (21.4)
50–75th	1117 (22.1)	1611 (20.1)	2604 (22.8)	41,357 (23.0)
≤75th (lowest)	1660 (32.8)	1998 (25.0)	4149 (36.3)	62,724 (34.9)
Unknown	314 (6.2)	825 (10.3)	621 (5.4)	10,118 (5.6)
**Personal urbanization level**				
Level 1 (urban)	2918 (57.6)	4381 (54.7)	6,520 (57.1)	100,857 (56.1)
Level 2	1785 (35.3)	2984 (37.3)	4,010 (35.1)	63,765 (35.5)
Level 3	278 (5.5)	481 (6.0)	680 (6.0)	11,000 (6.1)
Level 4 (rural)	52 (1.0)	81 (1.0)	118 (1.0)	1819 (1.0)
Unknown	30 (0.6)	75 (0.9)	99 (0.9)	2233 (1.2)
**Psychiatric hospitalizations**				
0	4590 (90.7)	7984 (99.8)	10,317 (90.3)	147,908 (82.3)
1	331 (6.5)	11 (0.1)	727 (6.4)	23,490 (13.1)
≥2	142 (2.8)	7 (0.1)	383 (3.4)	8276 (4.6)
**Psychiatric outpatient visits**				
0–6	3211 (63.4)	7841 (98.0)	7125 (62.4)	100,776 (56.1)
7–13	1008 (19.9)	107 (1.3)	2421 (21.2)	40,730 (22.7)
≥14	844 (16.7)	54 (0.7)	1881 (16.5)	38,168 (21.2)
**Medical comorbidities**				
0 (Charlson Comorbidity Index)	3979 (78.6)	7083 (88.5)	7450 (65.2)	110,760 (61.6)
1–2	964 (19.0)	852 (10.6)	3030 (26.5)	53,251 (29.6)
≥3	120 (2.4)	67 (0.8)	947 (8.3)	15,663 (8.7)
**Psychiatric comorbidities**				
Neurodevelopmental disorders	237 (4.7)	63 (0.8)	348 (3.0)	4463 (2.5)
Anxiety disorders	1086 (21.4)	144 (1.8)	2536 (22.2)	31,092 (17.3)
Obsessive-compulsive disorders	110 (2.2)	20 (0.2)	198 (1.7)	3581 (2.0)
Post-traumatic stress disorders	42 (0.8)	≤5 (<0.1)	89 (0.8)	947 (0.5)
Eating disorders	77 (1.5)	6 (0.1)	109 (1.0)	1320 (0.7)
Substance use disorders	217 (4.3)	14 (0.2)	561 (4.9)	5950 (3.3)
Personality disorders	116 (2.3)	5 (0.1)	217 (1.9)	3745 (2.1)
**Psychiatric medication use**				
Antidepressant use	353 (7.0)	11 (0.1)	813 (7.1)	14,281 (7.9)
Antipsychotic use	343 (6.8)	12 (0.1)	860 (7.5)	28,159 (15.7)
Mood stabilizer use	284 (5.6)	≤5 (<0.1)	624 (5.5)	22,352 (12.4)

[1] Data was expressed as mean ± standard deviation or N (percentage).

[2] In accordance with National Health Insurance Research Database regulations, cells with counts ≤5 were not reported to protect confidentiality.

© 2026 Hsu C-W et al. *JAMA Network Open*.

eTable 12. Risk of all-cause and cause-specific mortality in participants with bipolar II disorder and their unaffected siblings

Characteristics	Case, event (n = 5063)	Control, event (n = 8002)	Model 1 (Crude hazard ratio)	Model 2 (Adjusted hazard ratio)
**All-cause**	174 (3.4)	158 (2.0)	1.74 (1.41–2.15)	1.31 (1.003–1.72)
**Natural causes**	70 (1.4)	87 (1.1)	1.27 (0.94–1.73)	0.78 (0.51–1.17)
Certain infectious and parasitic diseases	0 (0.0)	4 (<0.1)	–	
Neoplasms	8 (0.2)	25 (0.3)	0.51 (0.23–1.09)	0.51 (0.22–1.18)
Diseases of the blood and blood-forming organs and certain disorders	1 (<0.1)	1 (<0.1)	1.58 (0.10–25.31)	–
Endocrine, nutritional, and metabolic diseases	10 (0.2)	4 (<0.1)	3.96 (1.25–12.61)	2.55 (0.75–8.67)
Mental and behavioral disorders	3 (0.1)	2 (<0.1)	2.38 (0.40–14.37)	0.38 (0.004–39.63)
Diseases of the nervous system	2 (<0.1)	4 (<0.1)	0.79 (0.14–4.31)	0.16 (0.08–0.32)
Diseases of the eye and adnexa	0 (0.0)	0 (0.0)	–	–
Diseases of the ear and mastoid process	0 (0.0)	0 (0.0)	–	–
Diseases of the circulatory system	16 (0.3)	12 (0.1)	2.11 (1.00–4.46)	1.43 (0.60–3.43)
Diseases of the respiratory system	6 (0.1)	3 (<0.1)	3.16 (0.79–12.67)	1.20 (0.20–7.27)
Diseases of the digestive system	10 (0.2)	16 (0.2)	0.99 (0.45–2.18)	0.51 (0.15–1.70)
Diseases of the skin and subcutaneous tissue	0 (0.0)	0 (0.0)	–	–
Diseases of the musculoskeletal system and connective tissue	2 (<0.1)	1 (<0.1)	3.16 (0.29–34.98)	1.69 (0.09–32.88)
Diseases of the genitourinary system	3 (0.1)	1 (<0.1)	4.79 (0.50–45.97)	1.32 (0.01–169.03)
Pregnancy, childbirth, and the puerperium	0 (0.0)	1 (<0.1)	–	–
Certain conditions originating in the perinatal period	0 (0.0)	4 (<0.1)	–	–
Congenital malformations, deformations and chromosomal abnormalities	0 (0.0)	2 (<0.1)	–	–
Symptoms, signs and abnormal clinical and laboratory findings, not elsewhere classified	9 (0.2)	7 (0.1)	2.03 (0.76–5.46)	0.72 (0.22–2.29)
**Unnatural causes**	104 (2.1)	70 (0.9)	2.35 (1.74–3.18)	2.05 (1.43–2.95)
Unintentional injuries	20 (0.4)	29 (0.4)	1.09 (0.62–1.93)	1.00 (0.50–2.00)
Suicide	81 (1.6)	38 (0.5)	3.37 (2.31–4.93)	2.83 (1.78–4.49)
Assault or homicide	3 (0.1)	3 (<0.1)	1.59 (0.32–7.86)	1.57 (0.40–6.19)
**Unknown causes**	0 (0.0)	1 (<0.1)	–	–

[1] Event was expressed as N (percentage).

[2] Model 2 adjusted for all variables (sex, birth year, income level, urbanization level, psychiatric healthcare utilization, Charlson Comorbidity Index, and birth order).

© 2026 Hsu C-W et al. *JAMA Network Open*.

eTable 13. Risk of all-cause and cause-specific mortality in participants with bipolar II disorder and participants with bipolar I disorder

Characteristics	Case, event (n = 11,427)	Control, event (n = 179,674)	Model 1 (Crude hazard ratio)	Model 2 (Adjusted hazard ratio)
**All-cause**	1089 (9.5)	29,177 (16.2)	0.95 (0.90–1.01)	1.24 (1.01–1.53)
**Natural causes**	793 (6.9)	21,878 (12.2)	0.96 (0.90–1.03)	1.45 (1.14–1.86)
Certain infectious and parasitic diseases	19 (0.2)	715 (0.4)	0.70 (0.45–1.11)	0.57 (0.08–4.27)
Neoplasms	174 (1.5)	4745 (2.6)	0.97 (0.83–1.13)	0.92 (0.47–1.80)
Diseases of the blood and blood-forming organs and certain disorders	4 (<0.1)	54 (<0.1)	1.95 (0.70–5.43)	13.33 (1.64–108.32)
Endocrine, nutritional, and metabolic diseases	68 (0.6)	2041 (1.1)	0.91 (0.71–1.16)	1.73 (0.86–3.50)
Mental and behavioral disorders	21 (0.2)	733 (0.4)	0.78 (0.51–1.21)	4.32 (1.79–10.42)
Diseases of the nervous system	22 (0.2)	676 (0.4)	0.91 (0.59–1.39)	1.94 (0.58–6.48)
Diseases of the eye and adnexa	0 (0.0)	1 (<0.1)	–	–
Diseases of the ear and mastoid process	0 (0.0)	1 (<0.1)	–	–
Diseases of the circulatory system	197 (1.7)	5379 (3)	0.97 (0.84–1.12)	1.42 (0.86–2.32)
Diseases of the respiratory system	108 (0.9)	3150 (1.8)	0.94 (0.77–1.14)	1.19 (0.56–2.55)
Diseases of the digestive system	77 (0.7)	1772 (1.0)	1.09 (0.87–1.37)	1.90 (0.99–3.65)
Diseases of the skin and subcutaneous tissue	4 (<0.1)	90 (0.1)	1.08 (0.40–2.94)	–
Diseases of the musculoskeletal system and connective tissue	10 (0.1)	206 (0.1)	1.25 (0.66–2.37)	5.07 (1.15–22.39)
Diseases of the genitourinary system	36 (0.3)	1048 (0.6)	0.94 (0.67–1.31)	1.37 (0.33–5.60)
Pregnancy, childbirth, and the puerperium	0 (0.0)	0 (0.0)	–	–
Certain conditions originating in the perinatal period	0 (0.0)	0 (0.0)	–	–
Congenital malformations, deformations and chromosomal abnormalities	1 (<0.1)	14 (<0.1)	1.68 (0.22–12.87)	–
Symptoms, signs and abnormal clinical and laboratory findings, not elsewhere classified	52 (0.5)	1253 (0.7)	1.01 (0.77–1.34)	0.70 (0.21–2.29)
**Unnatural causes**	288 (2.5)	7044 (3.9)	0.92 (0.82–1.04)	0.80 (0.54–1.18)
Unintentional injuries	81 (0.7)	2044 (1.1)	0.95 (0.76–1.19)	0.60 (0.26–1.40)
Suicide	203 (1.8)	4939 (2.7)	0.90 (0.79–1.04)	0.87 (0.56–1.36)
Assault or homicide	4 (<0.1)	61 (<0.1)	1.49 (0.54–4.10)	–
**Unknown causes**	8 (0.1)	255 (0.1)	1.16 (0.57–2.35)	–

[1] Event was expressed as N (percentage).

[2] Model 2 adjusted for all variables (sex, birth year, income level, urbanization level, psychiatric healthcare utilization, and Charlson Comorbidity Index).

© 2026 Hsu C-W et al. *JAMA Network Open*.

eFigure 1. Flowchart of the selection process for the study

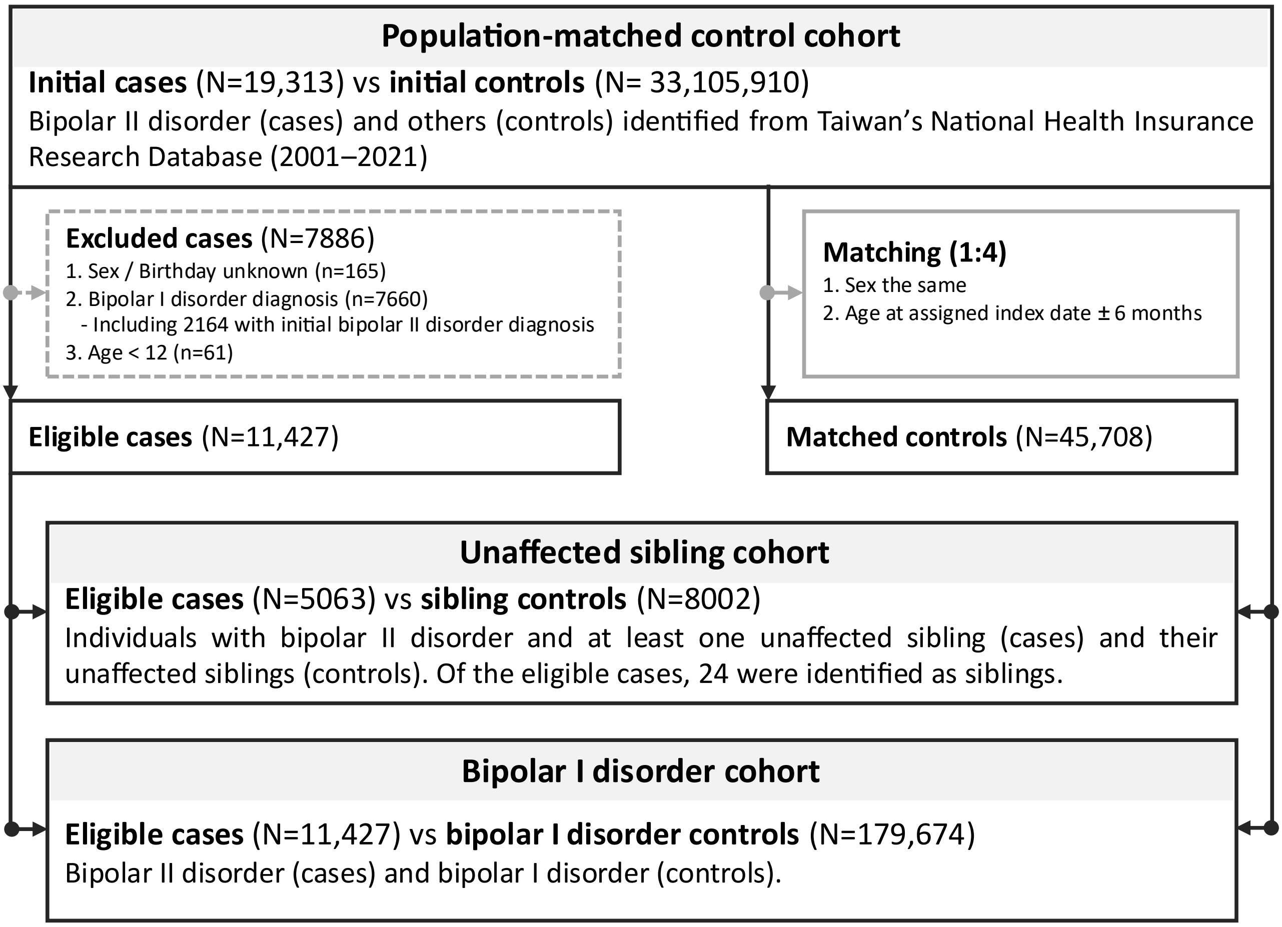

© 2026 Hsu C-W et al. *JAMA Network Open*.

eFigure 2. Kaplan-Meier survival curves for all-cause mortality in participants with bipolar II disorder (case) and matched controls (control)

(A) All-cause

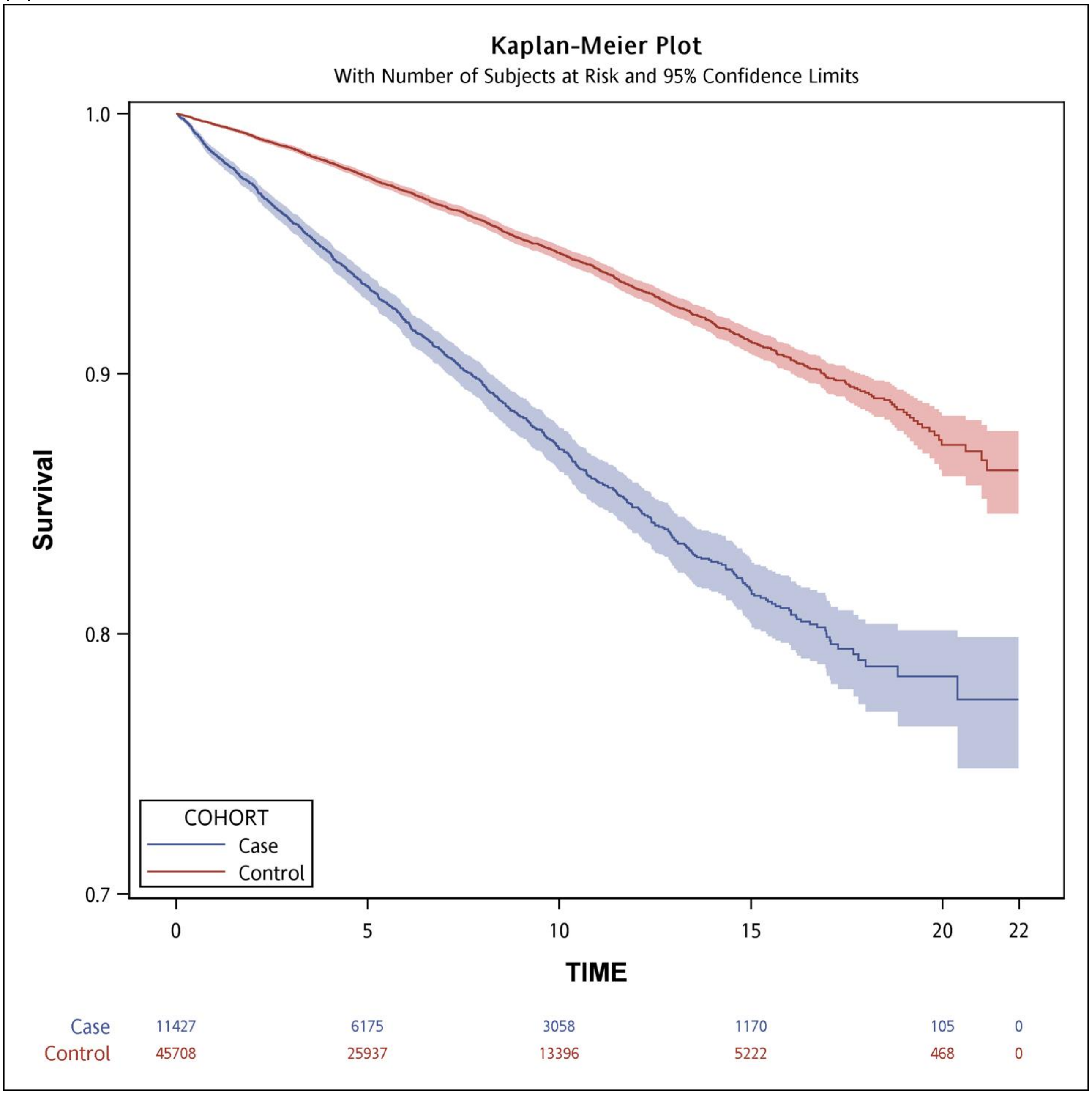

Log-rank test: P<0.001

© 2026 Hsu C-W et al. *JAMA Network Open*.

(B) Natural causes

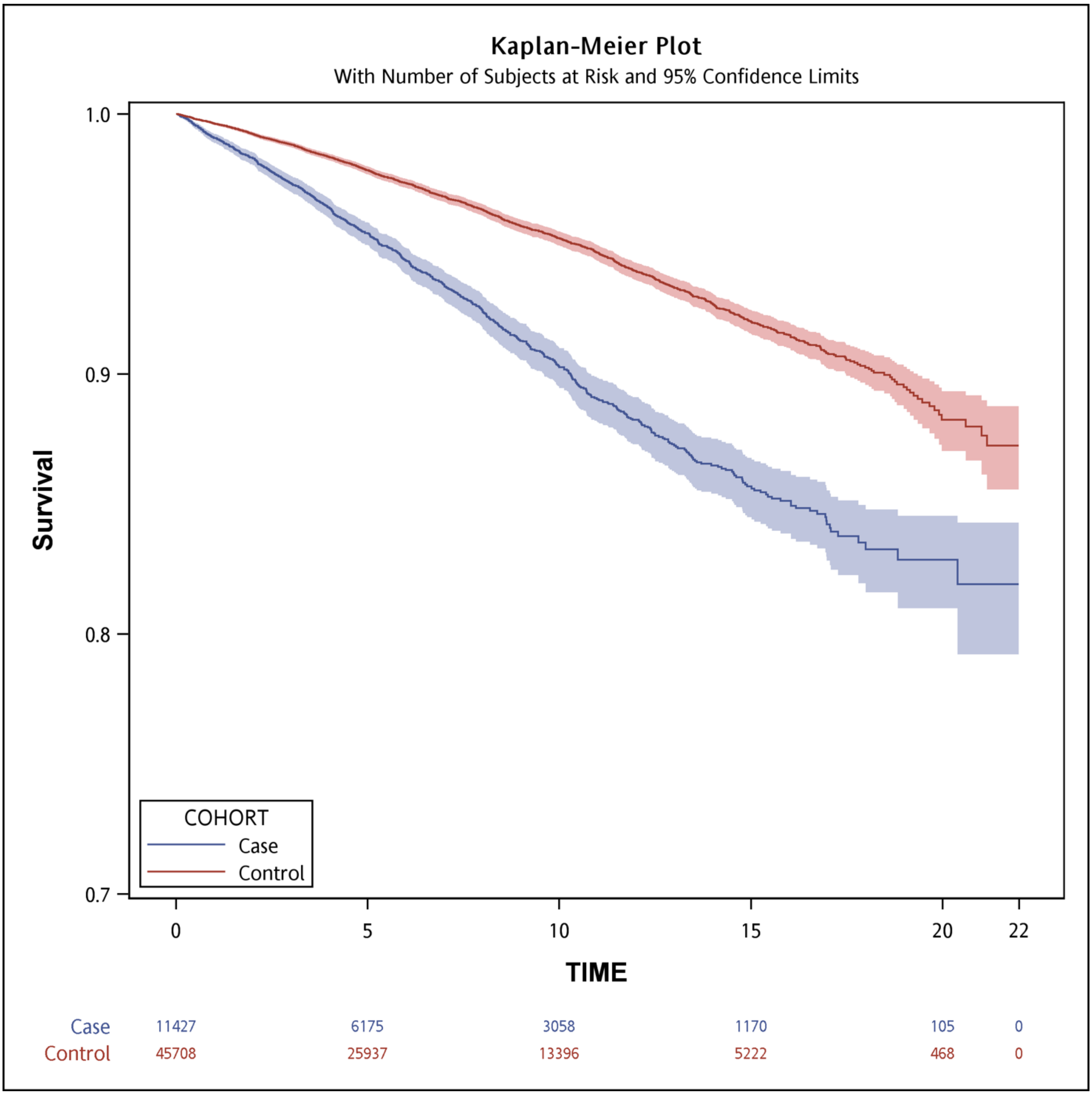

Log-rank test: P<0.001

© 2026 Hsu C-W et al. *JAMA Network Open*.

(C) Unnatural causes

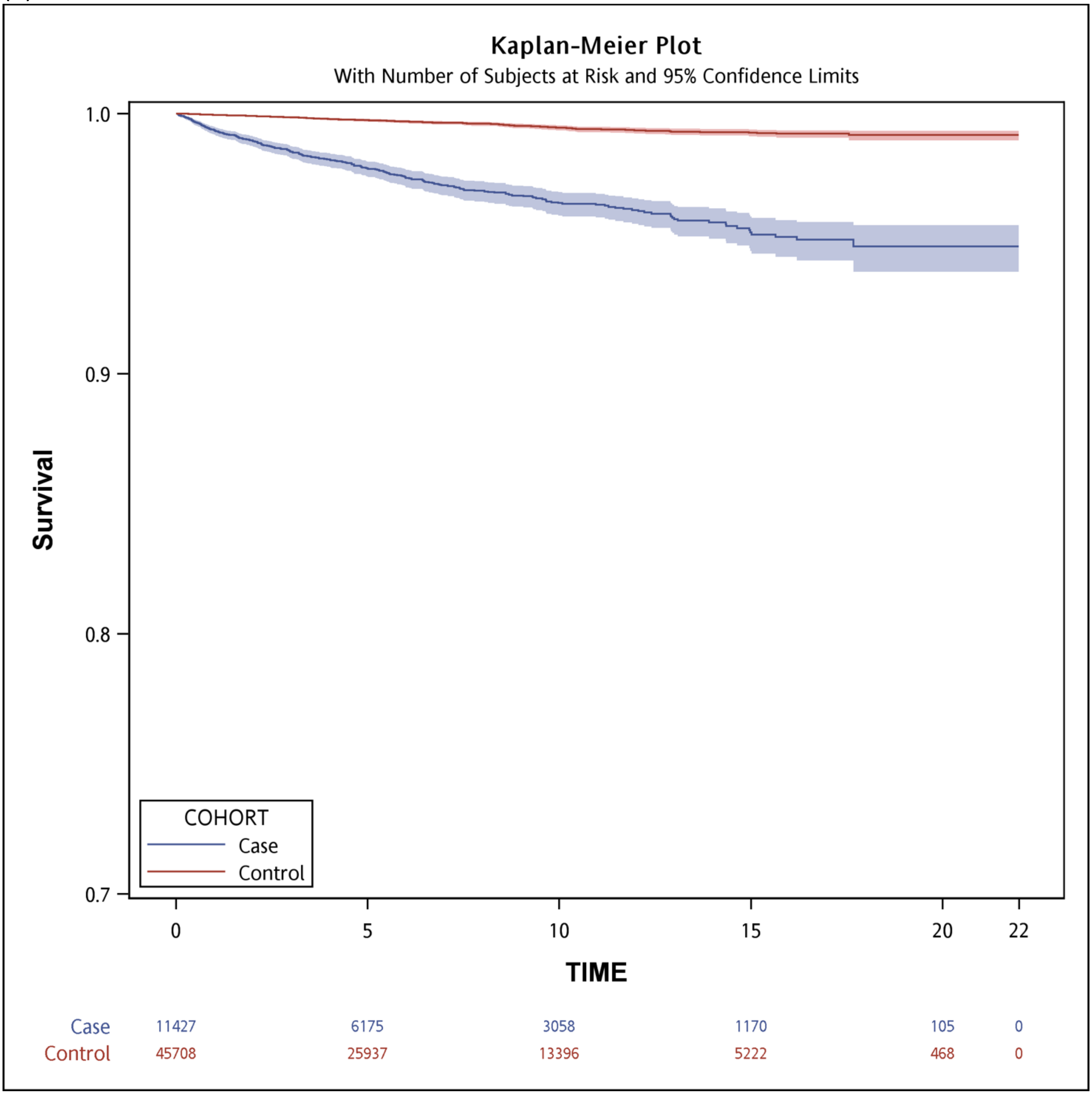

Log-rank test: P<0.001

© 2026 Hsu C-W et al. *JAMA Network Open*.

eReferences

1. Carvalho AF, Hsu CW, Vieta E, et al. Mortality and Lithium-Protective Effects after First-Episode Mania Diagnosis in Bipolar Disorder: A Nationwide Retrospective Cohort Study in Taiwan. *Psychother Psychosom*. 2024;93(1):36-45. doi:10.1159/000535777
2. Hsieh CY, Su CC, Shao SC, et al. Taiwan's National Health Insurance Research Database: past and future. *Clin Epidemiol*. 2019;11:349-358. doi:10.2147/clep.S196293
3. Bolton S, Warner J, Harriss E, Geddes J, Saunders KEA. Bipolar disorder: Trimodal age-at-onset distribution. *Bipolar Disord*. Jun 2021;23(4):341-356. doi:10.1111/bdi.13016
4. Tsai Y-H, Guan J-Y, Huang Y-H. Do Degradation of Urban Greenery and Increasing Land Prices Often Come along with Urbanization? In: Ergen M, ed. *Urban Agglomeration*. IntechOpen; 2017.
5. Charlson ME, Pompei P, Ales KL, MacKenzie CR. A new method of classifying prognostic comorbidity in longitudinal studies: development and validation. *J Chronic Dis*. 1987;40(5):373-83. doi:10.1016/0021-9681(87)90171-8
6. Catalá-López F, Hutton B, Page MJ, et al. Mortality in Persons With Autism Spectrum Disorder or Attention-Deficit/Hyperactivity Disorder: A Systematic Review and Meta-analysis. *JAMA Pediatr*. Apr 1 2022;176(4):e216401. doi:10.1001/jamapediatrics.2021.6401
7. Meier SM, Mattheisen M, Mors O, Mortensen PB, Laursen TM, Penninx BW. Increased mortality among people with anxiety disorders: total population study. *Br J Psychiatry*. Sep 2016;209(3):216-21. doi:10.1192/bjp.bp.115.171975
8. Fernández de la Cruz L, Isomura K, Lichtenstein P, et al. All cause and cause specific mortality in obsessive-compulsive disorder: nationwide matched cohort and sibling cohort study. *Bmj*. Jan 17 2024;384:e077564. doi:10.1136/bmj-2023-077564
9. Nilaweera D, Phyo AZZ, Teshale AB, et al. Lifetime posttraumatic stress disorder as a predictor of mortality: a systematic review and meta-analysis. *BMC Psychiatry*. Apr 10 2023;23(1):229. doi:10.1186/s12888-023-04716-w
10. Krug I, Liu S, Portingale J, et al. A meta-analysis of mortality rates in eating disorders: An update of the literature from 2010 to 2024. *Clin Psychol Rev*. Mar 2025;116:102547. doi:10.1016/j.cpr.2025.102547
11. Charlson FJ, Baxter AJ, Dua T, Degenhardt L, Whiteford HA, Vos T. Excess mortality from mental, neurological and substance use disorders in the Global Burden of Disease Study 2010. *Epidemiol Psychiatr Sci*. Apr 2015;24(2):121-40. doi:10.1017/s2045796014000687
12. Høye A, Jacobsen BK, Bramness JG, Nesvåg R, Reichborn-Kjennerud T, Heiberg I. Total and cause-specific mortality in patients with personality disorders: the association between comorbid severe mental illness and substance use disorders. *Soc Psychiatry Psychiatr Epidemiol*. Oct 2021;56(10):1809-1819. doi:10.1007/s00127-021-02055-3
13. Schisterman EF, Cole SR, Platt RW. Overadjustment bias and unnecessary adjustment in epidemiologic studies. *Epidemiology*. Jul 2009;20(4):488-95. doi:10.1097/EDE.0b013e3181a819a1
14. Tchetgen Tchetgen EJ, Phiri K. Evaluation of Medication-mediated Effects in Pharmacoepidemiology. *Epidemiology*. May 2017;28(3):439-445. doi:10.1097/ede.0000000000000610

© 2026 Hsu C-W et al. *JAMA Network Open*.

15. Colin Cameron A, Miller DL. A Practitioner’s Guide to Cluster-Robust Inference. *Journal of Human Resources*. 2015;50(2):317. doi:10.3368/jhr.50.2.317

© 2026 Hsu C-W et al. *JAMA Network Open*.
