# Supplementary material for: All-Cause and Cause-Specific Mortality in Patients With Bipolar II Disorder
Source: JAMA Netw Open. 2026 Apr 7;9(4):e265535. doi: 10.1001/jamanetworkopen.2026.5535 (PMC13058765; doi:10.1001/jamanetworkopen.2026.5535)
Supplement: Supplement 2. — Data Sharing Statement [file jamanetwopen-e265535-s002.pdf]

Data Sharing Statement

Hsu. All-Cause and Cause-Specific Mortality in Patients With Bipolar II Disorder. *JAMA Netw Open*. Published April 07, 2026. doi:10.1001/jamanetworkopen.2026.5535

Data
Data available: Yes
Data types: Data dictionary
How to access data: harwicacademia@gmail.com
When available: With publication

Supporting Documents
Document types: None

Additional Information
Who can access the data: researchers whose proposed use of the data has been approved
Types of analyses: for selected purposes
Mechanisms of data availability: with a signed data access agreement
Any additional restrictions: with permission from the Taiwan Ministry of Health and Welfare
